# Supplementary material for: Self-directed digital interventions for the improvement of emotion regulation—effectiveness for mental health and functioning in adolescents: protocol for a systematic review
Source: BMJ Open. 2024 Apr 23;14(4):e081556. doi: 10.1136/bmjopen-2023-081556 (PMC11043735; doi:10.1136/bmjopen-2023-081556)
Supplement: Supplementary data [file bmjopen-2023-081556supp001.pdf]

Supplementary File 1

Database: Ovid PsycINFO 1806 to present

Search Strategy:

----

1 (adolescen* or juvenil* or youth* or teen* or pubescen* or pediatric* or paediatric* or "high school" or "secondary school" or school* or "young people" or "young person*" or "young adult*" or "young man" or "young men" or "young woman" or "young women" or student*).ti,ab

2 (emotion* AND (regulat* or dysregulat* or generat* OR modulat* OR adapt* OR cop* OR react* OR arous* OR avoid* OR behavio* OR suppress* OR ruminat*)).,ti,ab

3 (affect AND (regulat* or dysregulat* or generat* OR modulat* OR adapt* OR cop* OR react* OR arous* OR avoid* OR behavio* OR suppress* OR ruminat*)).ti,ab

4 ("self-concept" OR "mood regulat*" OR "situation selection" OR "situation modification" OR "attentional deployment" OR "response modulation" OR "problem focused coping" OR "impulsivity" OR "hyper-reactivity" OR "anger regulation" OR "experiential avoidance" OR "expressive suppression" OR "thought suppression" OR "ruminat*" OR "emotion regulation questionnaire" OR "Difficulties in Emotion Regulation Scale" OR "emotion regulation strategy").ti,ab

5 2 or 3 or 4

6 (treat* or interven* or "early intervention" or "self-directed intervention" or "self-directed" or "public health").ti,ab.

7 phon* or mobile* or "smart-phone*" or device* or computer* or tablet* or "device-based" or "app-based" or apps or app or "sensor*" or "social media").ti,ab.

8 ((digital or virtual or online or interactive) and (health* or intervention or support or platform*)).ti,ab.

9 ("eHealth" or "e-health" or "m-health" or mHealth or "tele-health" or telehealth).ti,ab.

10 7 or 8 or 9

11 1 and 5 and 6 and 10

12 limit 11 to (peer reviewed journal and human and english language and (childhood <birth to 12 years> or adolescence <13 to 17 years>) and ("0100 journal" or "0110 peer-reviewed journal") and journal article and english and human and yr="1995 -Current")

Database: PubMed MEDLINE

Search Strategy:

----

1 adolescen*[Title/Abstract] OR juvenil*[Title/Abstract] OR youth*[Title/Abstract] OR teen*[Title/Abstract] OR pubescen*[Title/Abstract] OR pediatric*[Title/Abstract] OR paediatric*[Title/Abstract] OR "high school"[Title/Abstract] OR "secondary school"[Title/Abstract] OR school*[Title/Abstract] OR "young people"[Title/Abstract] OR "young person*"[Title/Abstract] OR "young adult*"[Title/Abstract] OR "young man"[Title/Abstract] OR "young men"[Title/Abstract] OR "young woman"[Title/Abstract] OR "young women"[Title/Abstract] OR student*[Title/Abstract]

2 emotion*[Title/Abstract] AND (regulat*[Title/Abstract] OR dysregulat*[Title/Abstract] OR generat*[Title/Abstract] OR modulat*[Title/Abstract] OR adapt*[Title/Abstract] OR react*[Title/Abstract] OR arous*[Title/Abstract] OR avoid*[Title/Abstract] OR behavio*[Title/Abstract] OR suppress*[Title/Abstract] OR ruminat*[Title/Abstract])

3 affect[Title/Abstract] AND (regulat*[Title/Abstract] OR dysregulat*[Title/Abstract] OR generat*[Title/Abstract] OR modulat*[Title/Abstract] OR adapt*[Title/Abstract] OR react*[Title/Abstract] OR arous*[Title/Abstract] OR avoid*[Title/Abstract] OR behavio*[Title/Abstract] OR suppress*[Title/Abstract] OR ruminat*[Title/Abstract])

4 "self-concept"[Title/Abstract] OR "mood regulat*"[Title/Abstract] OR "situation selection"[Title/Abstract] OR "situation modification"[Title/Abstract] OR "attentional deployment"[Title/Abstract] OR "response modulation"[Title/Abstract] OR "problem focused coping"[Title/Abstract] OR "impulsivity"[Title/Abstract] OR "hyper-reactivity"[Title/Abstract] OR "anger regulation"[Title/Abstract] OR "experiential avoidance"[Title/Abstract] OR "expressive suppression"[Title/Abstract] OR "thought suppression"[Title/Abstract] OR "ruminat*"[Title/Abstract] OR "emotion regulation questionnaire"[Title/Abstract] OR "Difficulties in Emotion Regulation Scale"[Title/Abstract] OR "emotion regulation strategy"[Title/Abstract]

5 #2 OR #3 OR #4

6 treat*[Title/Abstract] OR interven*[Title/Abstract] OR "early intervention"[Title/Abstract] OR "self-directed intervention"[Title/Abstract] OR "self-directed"[Title/Abstract] OR "public health"[Title/Abstract]

7 phon*[Title/Abstract] OR mobile*[Title/Abstract] OR "smart-phone*"[Title/Abstract] OR device*[Title/Abstract] OR computer*[Title/Abstract] OR tablet*[Title/Abstract] OR "device-based"[Title/Abstract] OR "app-based"[Title/Abstract] OR apps[Title/Abstract] OR app[Title/Abstract] OR "sensor*"[Title/Abstract] OR "social media"[Title/Abstract]

8 ((digital[Title/Abstract] OR virtual[Title/Abstract] OR online[Title/Abstract] OR interactive[Title/Abstract]) AND (health*[Title/Abstract] OR intervention[Title/Abstract] OR support[Title/Abstract] OR platform*[Title/Abstract]))

9	"eHealth"[Title/Abstract] OR "e-health"[Title/Abstract] OR "m-health"[Title/Abstract] OR mHealth[Title/Abstract] OR "tele-health"[Title/Abstract] OR telehealth[Title/Abstract]
10	#7 OR #8 OR #9
11	#1 and #5 and #6 and #10
**12**	**#1 and #5 and #6 and #10 Filters: Humans, English, MEDLINE, from 1995 – 2023**

Database: Scopus

Search Strategy:

(TITLE-ABS-KEY (adolescen* OR juvenil* OR youth* OR teen* OR pubescen* OR pediatric* OR paediatric* OR "high school" OR "secondary school" OR school* OR "young people" OR "young person*" OR "young adult*" OR "young man" OR "young men" OR "young woman" OR "young women" OR student*) AND TITLE-ABS-KEY ((emotion* AND (regulat* OR dysregulat* OR generat* OR modulat* OR adapt* OR cop* OR react* OR arous* OR avoid* OR behavio* OR suppress* OR ruminat*)) OR (affect AND (regulat* OR dysregulat* OR generat* OR modulat* OR adapt* OR cop* OR react* OR arous* OR avoid* OR behavio* OR suppress* OR ruminat*)) OR (self-concept OR "mood regulat*" OR "situation selection" OR "situation modification" OR "attentional deployment" OR "response modulation" OR "problem focused coping" OR impulsivity OR hyper-reactivity OR "anger regulation" OR "experiential avoidance" OR "expressive suppression" OR "thought suppression" OR ruminat* OR "emotion regulation questionnaire" OR "Difficulties in Emotion Regulation Scale" OR "emotion regulation strategy")) AND TITLE-ABS-KEY (treat* OR interven* OR "early intervention" OR "self-directed intervention" OR "self-directed" OR "public health") AND TITLE-ABS-KEY ((phon* OR mobile* OR "smart-phone*" OR device* OR computer* OR tablet* OR "device-based" OR "app-based" OR apps OR app OR "sensor*" OR "social media") OR ((digital OR virtual OR online OR interactive) AND (health* OR intervention OR support OR platform*)) OR ("eHealth" OR "e-health" OR "m-health" OR mhealth OR "tele-health" OR telehealth))) AND ORIG-LOAD-DATE AFT 20230203 AND (LIMIT-TO (SUBJAREA , "MEDI") OR LIMIT-TO (SUBJAREA , "SOCI") OR LIMIT-TO (SUBJAREA , "MULT") OR LIMIT-TO (SUBJAREA , "PSYC") OR LIMIT-TO (SUBJAREA , "NURS") OR LIMIT-TO (SUBJAREA , "HEAL")) AND (LIMIT-TO (DOCTYPE , "ar")) AND (LIMIT-TO (EXACTKEYWORD , "Human") OR LIMIT-TO (EXACTKEYWORD , "Humans") OR LIMIT-TO (EXACTKEYWORD , "Adolescent")) AND (LIMIT-TO (LANGUAGE , "English")) AND (EXCLUDE (SUBJAREA , "MEDI") OR EXCLUDE (SUBJAREA , "NEUR") OR EXCLUDE (SUBJAREA , "BIOC") OR EXCLUDE (SUBJAREA , "ARTS") OR EXCLUDE (SUBJAREA , "ENVI") OR EXCLUDE (SUBJAREA , "PHAR")) AND (EXCLUDE (SUBJAREA , "AGRI") OR EXCLUDE (SUBJAREA , "BUSI") OR EXCLUDE (SUBJAREA , "COMP") OR EXCLUDE (SUBJAREA , "DENT") OR EXCLUDE (SUBJAREA , "ENGI")) AND (EXCLUDE (SUBJAREA , "VETE"))

Database: Global Health 1973 to 2022 Week 50

Search Strategy:

----

1 (adolescen* or juvenil* or youth* or teen* or pubescen* or pediatric* or paediatric* or "high school" or "secondary school" or school* or "young people" or "young person*" or "young adult*" or "young man" or "young men" or "young woman" or "young women" or student*).ti,ab

2 (emotion* AND (regulat* or dysregulat* or generat* OR modulat* OR adapt* OR cop* OR react* OR arous* OR avoid* OR behavio* OR suppress* OR ruminat*)).,ti,ab

3 (affect AND (regulat* or dysregulat* or generat* OR modulat* OR adapt* OR cop* OR react* OR arous* OR avoid* OR behavio* OR suppress* OR ruminat*)).,ti,ab

4 ("self-concept" OR "mood regulat*" OR "situation selection" OR "situation modification" OR "attentional deployment" OR "response modulation" OR "problem focused coping" OR "impulsivity" OR "hyper-reactivity" OR "anger regulation" OR "experiential avoidance" OR "expressive suppression" OR "thought suppression" OR "ruminat*" OR "emotion regulation questionnaire" OR "Difficulties in Emotion Regulation Scale" OR "emotion regulation strategy").ti,ab

5 2 or 3 or 4

6 (treat* or interven* or "early intervention" or "self-directed intervention" or "self-directed" or "public health").ti,ab.

7 phon* or mobile* or "smart-phone*" or device* or computer* or tablet* or "device-based" or "app-based" or apps or app or "sensor*" or "social media").ti,ab.

8 ((digital or virtual or online or interactive) and (health* or intervention or support or platform*)).ti,ab.

9 ("eHealth" or "e-health" or "m-health" or mHealth or "tele-health" or telehealth).ti,ab.

10 7 or 8 or 9

11 1 and 5 and 6 and 10

12 limit 11 to (english language and english and (journal or journal article) and yr="1995 -Current")

Database: EBSCO CINAHL

Search Strategy:

1	AB adolescen* or juvenil* or youth* or teen* or pubescen* or pediatric* or paediatric* or "high school" or "secondary school" or school* or "young people" or "young person*" or "young adult*" or "young man" or "young men" or "young woman" or "young women" or student*
2	AB emotion* AND (regulat* or dysregulat* or generat* OR modulat* OR adapt* OR cop* OR react* OR arous* OR avoid* OR behavio* OR suppress* OR ruminat*)
3	AB affect AND (regulat* or dysregulat* or generat* OR modulat* OR adapt* OR cop* OR react* OR arous* OR avoid* OR behavio* OR suppress* OR ruminat*)
4	AB "self-concept" OR "mood regulat*" OR "situation selection" OR "situation modification" OR "attentional deployment" OR "response modulation" OR "problem focused coping" OR "impulsivity" OR "hyper-reactivity" OR "anger regulation" OR "experiential avoidance" OR "expressive suppression" OR "thought suppression" OR "ruminat*" OR “emotion regulation questionnaire” OR “Difficulties in Emotion Regulation Scale” OR “emotion regulation strategy”
5	S2 or S3 or S4
6	AB treat* or interven* or "early intervention" or "self-directed intervention" or "self-directed" or "public health"
7	AB phon* or mobile* or "smart-phone*" or device* or computer* or tablet* or "device-based" or "app-based" or apps or app or "sensor*" or "social media"
8	AB (digital or virtual or online or interactive) and (health* or intervention or support or platform*))
9	AB "eHealth" or "e-health" or "m-health" or mHealth or "tele-health" or telehealth
10	S7 or S8 or S9
**11**	**S1 and S5 and S6 and S10**
	**Limiters - Published Date: 19950101-; English Language; Peer Reviewed; Research Article; Human; Publication Type: Journal Article; Age Groups: Child: 6-12 years, Adolescent: 13-18 years; Language: English**

Database: EBSCO ERIC

Search Strategy:

----

1 AB adolescen* or juvenil* or youth* or teen* or pubescen* or pediatric* or paediatric* or "high school" or "secondary school" or school* or "young people" or "young person*" or "young adult*" or "young man" or "young men" or "young woman" or "young women" or student*

2 AB emotion* AND (regulat* or dysregulat* or generat* OR modulat* OR adapt* OR cop* OR react* OR arous* OR avoid* OR behavio* OR suppress* OR ruminat*)

3 AB affect AND (regulat* or dysregulat* or generat* OR modulat* OR adapt* OR cop* OR react* OR arous* OR avoid* OR behavio* OR suppress* OR ruminat*)

4 AB "self-concept” OR "mood regulat*" OR "situation selection" OR "situation modification" OR "attentional deployment" OR "response modulation" OR "problem focused coping" OR "impulsivity" OR "hyper-reactivity" OR "anger regulation" OR "experiential avoidance" OR "expressive suppression" OR "thought suppression" OR "ruminat*" OR “emotion regulation questionnaire” OR “Difficulties in Emotion Regulation Scale” OR “emotion regulation strategy”

5 S2 or S3 or S4

6 AB treat* or interven* or "early intervention" or "self-directed intervention" or "self-directed" or "public health"

7 AB phon* or mobile* or "smart-phone*" or device* or computer* or tablet* or "device-based" or "app-based" or apps or app or "sensor*" or "social media"

8 AB (digital or virtual or online or interactive) and (health* or intervention or support or platform*)

9 AB "eHealth" or "e-health" or "m-health" or mHealth or "tele-health" or telehealth

10 S7 or S8 or S9

11 S1 and S5 and S6 and S10

Limiters - Published Date: 19950101-; English Language; Peer Reviewed; Research Article; Human; Publication Type: Journal Article; Age Groups: Child: 6-12 years, Adolescent: 13-18 years; Language: English

Database: The Cochrane Central Register of Controlled Trials (CENTRAL)

Search Strategy:

----

1 adolescen* or juvenil* or youth* or teen* or pubescen* or pediatric* or paediatric* or "high school" or "secondary school" or school* or "young people" or "young person*" or "young adult*" or "young man" or "young men" or "young woman" or "young women" or student*

2 emotion* AND (regulat* or dysregulat* or generat* OR modulat* OR adapt* OR cop* OR react* OR arous* OR avoid* OR behavio* OR suppress* OR ruminat*)

3 affect AND (regulat* or dysregulat* or generat* OR modulat* OR adapt* OR cop* OR react* OR arous* OR avoid* OR behavio* OR suppress* OR ruminat*)

4 self-concept OR "mood regulat*" OR "situation selection" OR "situation modification" OR "attentional deployment" OR "response modulation" OR "problem focused coping" OR "impulsivity" OR hyper-reactivity OR "anger regulation" OR "experiential avoidance" OR "expressive suppression" OR "thought suppression" OR ruminat* OR “emotion regulation questionnaire” OR “Difficulties in Emotion Regulation Scale” OR “emotion regulation strategy”

5 #2 or #3 or #4

6 treat* or interven* or "early intervention" or "self-directed intervention" or "self-directed" or "public health"

7 phon* or mobile* or "smart-phone*" or device* or computer* or tablet* or "device-based" or "app-based" or apps or app or "sensor*" or "social media"

8 (digital or virtual or online or interactive) and (health* or intervention or support or platform*)

9 "eHealth" or "e-health" or "m-health" or mHealth or "tele-health" or telehealth

10 #7 or #8 or #9

11 #1 and #5 and #6 and #10

with Publication Year from 1995 to 2023, in Trials with Public Health, Developmental, Psychosocial and Learning Problems, Child Health in Cochrane Groups

Database: Web of Science: Core Collection

Search Strategy:

----

1 AB=(adolescen* or juvenil* or youth* or teen* or pubescen* or pediatric* or paediatric* or "high school" or "secondary school" or school* or "young people" or "young person*" or "young adult*" or "young man" or "young men" or "young woman" or "young women" or student*)

2 AB=(emotion* AND (regulat* or dysregulat* or generat* OR modulat* OR adapt* OR cop* OR react* OR arous* OR avoid* OR behavio* OR suppress* OR ruminat*))

3 AB=(affect* AND (regulat* or dysregulat* or generat* OR modulat* OR adapt* OR cop* OR react* OR arous* OR avoid* OR behavio* OR suppress* OR ruminat*))

4 AB=(self-concept OR "mood regulat*" OR "situation selection" OR "situation modification" OR "attentional deployment" OR "response modulation" OR "problem focused coping" OR "impulsivity" OR hyper-reactivity OR "anger regulation" OR "experiential avoidance" OR "expressive suppression" OR "thought suppression" OR ruminat* OR “emotion regulation questionnaire” OR “Difficulties in Emotion Regulation Scale” OR “emotion regulation strategy”)

5 #2 OR #3 OR #4

6 AB=(treat* or interven* or "early intervention" or "self-directed intervention" or "self-directed" or "public health")

7 AB=(phon* or mobile* or "smart-phone*" or device* or computer* or tablet* or "device-based" or "app-based" or apps or app or "sensor*" or "social media")

8 AB=((digital or virtual or online or interactive) and (health* or intervention or support or platform*))

9 AB=("eHealth" or "e-health" or "m-health" or mHealth or "tele-health" or telehealth)

10 #9 OR #8 OR #7

11 #10 AND #6 AND #5 AND #1

18 #17 and 2023 or 2022 or 2021 or 2020 or 2019 or 2000 or 2001 or 2002 or 2003 or 2004 or 2005 or 2006 or 2007 or 2008 or 2009 or 2010 or 2011 or 2012 or 2013 or 2014 or 2015 or 2016 or 2017 or 2018 (Publication Years) and English (Languages) and English (Languages) and Article (Document Types)

19 #10 AND #6 AND #5 AND #1 and 2023 or 2022 or 2021 or 2020 or 2019 or 2018 or 2017 or 2016 or 2015 or 2014 or 2013 or 2012 or 2011 or 2001 or 2002 or 2003 or 2004 or 2006 or 2007 or 2009 or 2010 or 2005 or 2008 or 1995 or 1996 or 1997 or 1998 or 1999 or 2000 (Publication Years) and Psychiatry or Public Environmental Occupational Health or Education Educational Research or Psychology Multidisciplinary or Psychology Clinical or Pediatrics or Health Care Sciences Services or Psychology Developmental or Psychology or Family Studies or Multidisciplinary Sciences or Nursing or Medicine Research Experimental or Medicine General Internal or Psychology Educational or Behavioral Sciences or Education Scientific Disciplines or Health Policy Services or Social Sciences Interdisciplinary or Social Work or Education Special or Psychology Social or Psychology Applied or Psychology Experimental or Sociology (Web of Science Categories) and

Book Review or Data Paper or Reprint or Editorial Material or Book Chapters or Biographical-Item or Review Article or Proceeding Paper or Early Access (Exclude – Document Types) and English (Languages) and Psychology or Psychiatry or Public Environmental Occupational Health or Health Care Sciences Services or Education Educational Research or Pediatrics or General Internal Medicine or Nursing or Family Studies or Science Technology Other Topics or Research Experimental Medicine or Behavioral Sciences or Social Sciences Other Topics or Social Work (Research Areas)

Database: Elsevier Embase 1947 to present

Search Strategy:

1	(adolescen* or juvenil* or youth* or teen* or pubescen* or pediatric* or paediatric* or "high school" or "secondary school" or school* or "young people" or "young person*" or "young adult*" or "young man" or "young men" or "young woman" or "young women" or student*).ti,ab
2	(emotion* AND (regulat* or dysregulat* or generat* OR modulat* OR adapt* OR cop* OR react* OR arous* OR avoid* OR behavio* OR suppress* OR ruminat*)).,ti,ab
3	(affect AND (regulat* or dysregulat* or generat* OR modulat* OR adapt* OR cop* OR react* OR arous* OR avoid* OR behavio* OR suppress* OR ruminat*)).,ti,ab
4	("self-concept" OR "mood regulat*" OR "situation selection" OR "situation modification" OR "attentional deployment" OR "response modulation" OR "problem focused coping" OR "impulsivity" OR "hyper-reactivity" OR "anger regulation" OR "experiential avoidance" OR "expressive suppression" OR "thought suppression" OR "ruminat*" OR "emotion regulation questionnaire" OR "Difficulties in Emotion Regulation Scale" OR "emotion regulation strategy").ti,ab
5	2 or 3 or 4
6	(treat* or interven* or "early intervention" or "self-directed intervention" or "self-directed" or "public health").ti,ab.
7	phon* or mobile* or "smart-phone*" or device* or computer* or tablet* or "device-based" or "app-based" or apps or app or "sensor*" or "social media").ti,ab.
8	((digital or virtual or online or interactive) and (health* or intervention or support or platform*)).ti,ab.
9	("eHealth" or "e-health" or "m-health" or mHealth or "tele-health" or telehealth).ti,ab.
10	7 or 8 or 9
11	1 and 5 and 6 and 10
**12**	**limit 11 to (human and english language and "remove medline records" and embase and yr="1995 -Current" and journal)**

Database: EThOS (PhD Theses)

Search Strategy:

1	"adolescen\" OR "high school" OR "secondary school" OR "youth" OR "young person" OR "young people"	Any word
2	AND "emotional regulation" OR "mood regulat\"	Any word
3	OR "emotion\" AND ("dysregulat\" OR "generat\" OR "modulat\" OR "adapt\" OR "cop\" OR react\ OR arous\ OR avoid\ OR behavio\ OR suppress\" OR "ruminat\")	Any word
4	OR affect AND (regulat\ or dysregulat\ or generat\ OR modulat\ OR adapt\ OR cop\ OR react\ OR arous\ OR avoid\ OR behavio\ OR "suppress\" OR "ruminat\”)	Any word
5	AND "prevent\" OR "promot\" OR "treat\" OR "evaluat\" OR "effective\" OR "interven\" OR "early intervention" OR "public health" OR "therap\"	Any word
6	AND "telemedicine\" OR "e-health\" OR "app-based" OR "virtual\" OR "web-based" OR "online\" OR "wearable" OR "device" OR "digital" OR "remote"	Any word

Database: OATD (PhD Theses)

Search Strategy:

1	"adolescen*" OR "youth*" OR "high school" OR "secondary school" OR "young person" OR "young people"	Any word
2	AND ("emotional regulation" OR "mood regulat*") OR ("emotion*" AND ("dysregulat*" OR "generat*" OR "modulat*" OR "adapt*" OR "cop*" OR react* OR arous* OR avoid* OR behavio* OR suppress*" OR "ruminat*")) OR (affect AND (regulat* or dysregulat* or generat* OR modulat* OR adapt* OR cop* OR react* OR arous* OR avoid* OR behavio* OR "suppress*" OR "ruminat*"))	Any word
3	AND "prevent*" OR "promot*" OR "treat*" OR "evaluat*" OR "effective*" OR "interven*" OR "early intervention" OR "public health" OR "therap*"	Any word
4	AND "telemedicine/" OR "e-health/" OR "app-based" OR "virtual*" OR "web-based" OR "online*" OR "wearable" OR "device" OR "digital" OR "remote"	Any word

Database: HMIC Health Management Information Consortium *1979 to November 2022*

Search Strategy:

----

1 exp adolescents/ or adolescent development/ or young adults/ or youth/

2 (adolescen* or juvenil* or youth* or teen* or pubescen* or pediatric* or paediatric* or "high school" or "secondary school" or school* or "young people" or "young person*" or "young adult*" or "young man" or "young men" or "young woman" or "young women" or student*).ti,ab.

3 1 or 2

4 “emotional regulation”.mp.

5 (emotion* AND (regulat* or dysregulat* or generat* OR modulat* OR adapt* OR cop* OR react* OR arous* OR avoid* OR behavio* OR suppress* OR ruminat*)).ti,ab.

6 (affect AND (regulat* or dysregulat* or generat* OR modulat* OR adapt* OR cop* OR react* OR arous* OR avoid* OR behavio* OR suppress* OR ruminat*)).ti,ab

7 ("self-concept" OR "mood regulat*" OR "situation selection" OR "situation modification" OR "attentional deployment" OR "response modulation" OR "problem focused coping" OR impulsivity OR "hyper-reactivity" OR "anger regulation" OR "experiential avoidance" OR "behavio* avoidance" OR "expressive suppression" OR "thought suppression" OR ruminat* OR “emotion regulation questionnaire” OR “Difficulties in Emotion Regulation Scale” OR “emotion regulation strategy”).ti,ab.

8 4 or 5 or 6 or 7

9 (prevent* or promot* or treat* or evaluat* or effective*).ti,ab.

10 (interven* or "early intervention" or "public health" or therap*).ti,ab.

11 9 or 10

12 telemedicine/ or e-health/

13 (phon* or mobile* or "smart-phone*" or device* or computer* or tablet* or "device-based" or "app-based" or apps or app or "sensor*" or "smart-home*" or "social media").ti,ab.

14 (tele and (phone* or nursing or health* or medicine or intervention or informatics or care or support or monitor* or presence or consultat* or rehab* or communicat*)).ti,ab.

15 (digital and (nursing* or health* or medicine or intervention or informatics or care or support or monitor* or presence or consultat* or rehab* or communicat* or platform*)).ti,ab.

16 (virtual and (nursing* or health* or medicine or intervention or informatics or care or support or monitor* or presence or consultat* or rehab* or communicat* or reality* or platform*)).ti,ab.

17 (online and (nursing* or health* or medicine or intervention or informatics or care or support or monitor* or presence or consultat* or rehab* or communicat* or platform*)).ti,ab

18	(interactiv* or "eHealth" or "e-health" or "e-therapy" or "e-learn" or "m-health").ti,ab.
19	("wireless health" or "health telematics" or "healthcare technology" or "therapeutic technology" or online or "medical informatics" or "medical information system" or "electronic health information" or "mobile application*" or "web-based" or website or wifi or "internet-delivered" or "internet-based" or "smart toy" OR "wearable*").ti,ab.
20	12 or 13 or 14 or 15 or 16 or 17 or 18 or 19
21	3 and 8 and 11 and 20
**22**	**limit 21 to (English language and yr="2000-2022")**

Database: PsyArXiv

Search Strategy:

("emotional regulation" OR "mood regulat*" OR "emotion dysregulat*" OR "arous*" OR "avoid*" OR "behavio*" OR "suppress*" OR "ruminat*") AND ("prevent*" OR "promot*" OR "treat*" OR "evaluat*" OR "effective*" OR "interven*" OR "early intervention" OR "public health" OR "therap*") AND ("telemedicine" OR "e-health" OR "app-based" OR "virtual*" OR "web-based" OR "online*" OR "wearable" OR "device" OR "digital" OR "remote")

Database: TRIP

Search Strategy:

("adolescen*" OR "youth*" OR "high school" OR "secondary school" OR "young person" OR "young people") AND ("emotional regulation" OR "mood regulat*" OR "emotion dysregulat*" OR "arous*" OR "avoid*" OR "behavio*" OR "suppress*" OR "ruminat*") AND ("prevent*" OR "promot*" OR "treat*" OR "evaluat*" OR "effective*" OR "interven*" OR "early intervention" OR "public health" OR "therap*") AND ("telemedicine" OR "e-health" OR "app-based" OR "virtual*" OR "web-based" OR "online*" OR "wearable" OR "device" OR "digital" OR "remote")

Database: ClinicalTrials.gov

Search Strategy:

("telemedicine" OR "e-health" OR "virtual*" OR "digital") | Emotional Regulation | Child

Database: ACM Digital Library

Search Strategy:

[[All: "emotional regulation"] OR [All: "mood regulat*"] OR [All: "emotion dysregulat*"]] AND [[All: "prevent*"] OR [All: "promot*"] OR [All: "treat*"] OR [All: "evaluat*"] OR [All: "effective*"] OR [All: "interven*"] OR [All: "early intervention"] OR [All: "public health"] OR [All: "therap*"]] AND [[All: "adolescen*"] OR [All: "youth*"] OR [All: "high school"] OR [All: "secondary school"] OR [All: "young person"] OR [All: "young people"]] AND [[All: "app-based"] OR [All: "virtual*"] OR [All: "web-based"] OR [All: "online*"] OR [All: "digital"] OR [All: "remote"]]

PRISMA-P (Preferred Reporting Items for Systematic review and Meta-Analysis Protocols) 2015 checklist: recommended items to address in a systematic review protocol*

Section and topic		Item No	Checklist item
**ADMINISTRATIVE INFORMATION**			
Title:			
Identification	✓	1a	Identify the report as a protocol of a systematic review
Update	N/A	1b	If the protocol is for an update of a previous systematic review, identify as such
Registration	✓	2	If registered, provide the name of the registry (such as PROSPERO) and registration number
Authors:			
Contact	✓	3a	Provide name, institutional affiliation, e-mail address of all protocol authors; provide physical mailing address of corresponding author
Contributions	✓	3b	Describe contributions of protocol authors and identify the guarantor of the review
Amendments	✓	4	If the protocol represents an amendment of a previously completed or published protocol, identify as such and list changes; otherwise, state plan for documenting important protocol amendments
Support:			
Sources	✓	5a	Indicate sources of financial or other support for the review
Sponsor	✓	5b	Provide name for the review funder and/or sponsor
Role of sponsor or funder	✓	5c	Describe roles of funder(s), sponsor(s), and/or institution(s), if any, in developing the protocol
**INTRODUCTION**			
Rationale	✓	6	Describe the rationale for the review in the context of what is already known
Objectives	✓	7	Provide an explicit statement of the question(s) the review will address with reference to participants, interventions, comparators, and outcomes (PICO)
**METHODS**			
Eligibility criteria	✓	8	Specify the study characteristics (such as PICO, study design, setting, time frame) and report characteristics (such as years considered, language, publication status) to be used as criteria for eligibility for the review
Information sources	✓	9	Describe all intended information sources (such as electronic databases, contact with study authors, trial registers or other grey literature sources) with planned dates of coverage
Search strategy	✓	10	Present draft of search strategy to be used for at least one electronic database, including planned limits, such that it could be repeated
Study records:			
Data management	✓	11a	Describe the mechanism(s) that will be used to manage records and data throughout the review

Selection process	✓	11b	State the process that will be used for selecting studies (such as two independent reviewers) through each phase of the review (that is, screening, eligibility and inclusion in meta-analysis)
Data collection process	✓	11c	Describe planned method of extracting data from reports (such as piloting forms, done independently, in duplicate), any processes for obtaining and confirming data from investigators
Data items	✓	12	List and define all variables for which data will be sought (such as PICO items, funding sources), any pre-planned data assumptions and simplifications
Outcomes and prioritization	✓	13	List and define all outcomes for which data will be sought, including prioritization of main and additional outcomes, with rationale
Risk of bias in individual studies	✓	14	Describe anticipated methods for assessing risk of bias of individual studies, including whether this will be done at the outcome or study level, or both; state how this information will be used in data synthesis
Data synthesis	✓	15a	Describe criteria under which study data will be quantitatively synthesised
	✓	15b	If data are appropriate for quantitative synthesis, describe planned summary measures, methods of handling data and methods of combining data from studies, including any planned exploration of consistency (such as I^2, Kendall's τ)
	✓	15c	Describe any proposed additional analyses (such as sensitivity or subgroup analyses, meta-regression)
	✓	15d	If quantitative synthesis is not appropriate, describe the type of summary planned
Meta-bias(es)	✓	16	Specify any planned assessment of meta-bias(es) (such as publication bias across studies, selective reporting within studies)
Confidence in cumulative evidence	✓	17	Describe how the strength of the body of evidence will be assessed (such as GRADE)

*** It is strongly recommended that this checklist be read in conjunction with the PRISMA-P Explanation and Elaboration (cite when available) for important clarification on the items. Amendments to a review protocol should be tracked and dated. The copyright for PRISMA-P (including checklist) is held by the PRISMA-P Group and is distributed under a Creative Commons Attribution Licence 4.0.**

From: Shamseer L, Moher D, Clarke M, Ghersi D, Liberati A, Petticrew M, Shekelle P, Stewart L, PRISMA-P Group. Preferred reporting items for systematic review and meta-analysis protocols (PRISMA-P) 2015: elaboration and explanation. BMJ. 2015 Jan 2;349(jan02 1):g7647.
