# Supplementary material for: Self-directed digital interventions for the improvement of emotion regulation—effectiveness for mental health and functioning in adolescents: protocol for a systematic review
Source: BMJ Open. 2024 Apr 23;14(4):e081556. doi: 10.1136/bmjopen-2023-081556 (PMC11043735; doi:10.1136/bmjopen-2023-081556)
Supplement: Author's manuscript [file bmjopen-2023-081556.draft_revisions.pdf]

BMJ Open

BMJ Open is committed to open peer review. As part of this commitment we make the peer review history of every article we publish publicly available.

When an article is published we post the peer reviewers' comments and the authors' responses online. We also post the versions of the paper that were used during peer review. These are the versions that the peer review comments apply to.

The versions of the paper that follow are the versions that were submitted during the peer review process. They are not the versions of record or the final published versions. They should not be cited or distributed as the published version of this manuscript.

BMJ Open is an open access journal and the full, final, typeset and author-corrected version of record of the manuscript is available on our site with no access controls, subscription charges or pay-per-view fees (http://bmjopen.bmj.com).

If you have any questions on BMJ Open's open peer review process please email info.bmjopen@bmj.com

BMJ Open

Self-Directed Digital Interventions for the Improvement of Emotion Regulation - Effectiveness for Mental Health and Functioning in Adolescents: Protocol for a Systematic Review

Journal:	*BMJ Open*
Manuscript ID	bmjopen-2023-081556
Article Type:	Protocol
Date Submitted by the Author:	31-Oct-2023
Complete List of Authors:	Thomson, Abigail; Queen Mary University of London, Centre for Psychiatry and Mental Health, Wolfson Institute of Population Health<br>Lawrence, Erin; Queen Mary University of London, Centre for Psychiatry and Mental Health, Wolfson Institute of Population Health<br>Oliver, Bonamy; University College London, UCL Institute of Education<br>Wright, Ben; East London NHS Foundation Trust; City University of London<br>Hosang, Georgina M.; Queen Mary University of London, Centre for Psychiatry and Mental Health, Wolfson Institute of Population Health
Keywords:	Adolescents < Adolescent, MENTAL HEALTH, eHealth

SCHOLARONE™
Manuscripts

Review Protocol

Title: Self-Directed Digital Interventions for the Improvement of Emotion Regulation - Effectiveness for Mental Health and Functioning in Adolescents: Protocol for a Systematic Review

ABSTRACT

Introduction

Emerging evidence has shown that problems with emotion regulation – how a person manages and responds to an emotional experience — are related to a range of psychological disorders, including eating disorders, bipolar disorder, anxiety, and depression. Interventions targeting emotion regulation processes have been shown to improve mental health in adults, but evidence on related interventions for adolescents is scarce. There is increasing interest in self-directed digital interventions that target emotion regulation. Still, questions remain about their effectiveness in supporting adolescents' mental health and overall functioning (e.g. academic achievement).

Methods and Analysis

Systematic searches will be conducted in the electronic databases MEDLINE, PsycINFO, Global Health, Scopus, Web of Science Core Collection, EBSCO CINAHL, EBSCO ERIC, OVID Embase, and The Cochrane Central Register of Controlled Trials (CENTRAL) to identify all studies examining self-directed digital interventions for adolescents, which include an emotion (dys-) regulation component. Grey literature, such as preprints and theses, are also included in this review. Data on the study characteristics (e.g. author(s)) and methodology, participant characteristics (e.g. age) and the digital interventions used to address emotion (dys-)regulation (e.g. name, focus) will be synthesised. A summary of observed changes in emotion (dys-)regulation or psychopathology occurring as a result of the intervention will be provided. Additionally, any general improvements in functioning (e.g. social) that occur as a result of participation in the intervention will also be reported if described in the included studies. Study quality will be assessed with the Effective Public Health Practice Project quality assessment tool. A narrative synthesis of all studies will be presented. If feasible, the effectiveness data will be synthesised using appropriate statistical techniques.

Ethics and Dissemination

Ethical approval is not required for this study. Findings will be disseminated widely via peer-reviewed publications and presentations at conferences related to this field.

For peer review only

Registration Details

PROSPERO CRD42022385547

STRENGTHS AND LIMITATIONS

- This review applies a systematic methodology that includes a comprehensive search strategy, duplicate screening, and quality assessment using validated tools
- Our results will provide up-to-date evidence on self-directed digital interventions for adolescent emotion (dys-)regulation and mental health, which are known to be sustainable, scalable, and engaging for adolescents
- By highlighting the most important components of these interventions, these results will also offer guidance for clinicians and researchers developing or delivering these interventions in practice, improving the quality of support offered to adolescents.
- This additional outcome has health economic benefits, ensuring that resources are allocated to those intervention components most effective in treating emotion dysregulation and/or psychopathology and functioning in adolescents.
- The heterogeneity of the interventions and tools used to measure emotion regulation may not allow for direct comparisons between studies

For peer review only

For peer review only - http://bmjopen.bmj.com/site/about/guidelines.xhtml

INTRODUCTION

Approximately 75% of mental health problems begin in adolescence [1]. In the UK, 1 in 6 adolescents (aged 11-16 years) have been identified as having a probable mental health problem – a figure which has steadily increased since 2004 [2]. Current attempts to support this population are designed to target specific conditions (e.g., depression). However, 60% of those adolescents with one diagnosable mental health problem have one or more additional conditions [3].

Mental health comorbidity – the presence of two or more mental illnesses in an individual - is the rule, rather than the exception, and has been associated with greater clinical severity and a greater detriment to overall quality of life [4]. There is also significant overlap in the aetiological origins of different psychopathologies, including evidence of major overlap in both genetic and environmental contributors between most disorders (e.g., major depressive disorder, bipolar disorder, and schizophrenia) [5]. Further complexity emerges from evidence linking non-specific risk factors (such as childhood adverse experiences: ACEs) with the onset of psychopathology [6]. ACEs (e.g., abuse and domestic violence) are a robust predictor of various mental and physical disorders and symptoms [7,8]. Whilst the mechanisms underpinning the aetiology and development of different psychopathologies are complex, associations have been found across a range of different conditions, including mood disorders, anxiety disorders, and substance use disorders [6].

A Transdiagnostic Approach

Given this, researchers have highlighted the need to understand and develop interventions that are directly effective across several disorders, or which alter psychopathological processes common to multiple conditions; these transdiagnostic strategies would offer broader, more effective support for adolescents struggling with their mental health [9]. The Unified Protocol for the transdiagnostic treatment of emotional disorders (UP) is one such strategy, which targets the emotional processes (e.g., emotional reactivity, emotional responding) underlying multiple different disorders. The UP is a transdiagnostic, emotion-focused cognitive-behavioural therapy (CBT) designed to treat all anxiety and unipolar mood disorders, as well as other disorders with strong emotional components (e.g., dissociative and somatoform disorders [10]). Preliminary evidence has found it an effective treatment for adolescents with emotional disorders [11, 12], demonstrating the possible utility of transdiagnostic approaches in adolescent mental health.

Emerging evidence suggests the transdiagnostic approach could be effective in targeting a range of psychopathologies in young people and can activate a range of related, beneficial developmental cascades, including improvement in social and academic outcomes in adolescence, as well as improved sleeping patterns [13]. A transdiagnostic approach to treatment is also thought to be time-

For peer review only - http://bmjopen.bmj.com/site/about/guidelines.xhtml

and cost-effective compared to disorder-specific strategies and may offer a more sustainable alternative to treatments currently available to this population [4].

Emotion Regulation – A Transdiagnostic Mechanism and Clinical Target?

Emotion regulation has received increased attention in recent years as a transdiagnostic mechanism and clinical target in psychological treatment [14]. Emotion regulation is a multidimensional process wherein an individual monitors, evaluates and shapes their emotions, when they have them, and how they experience or express them. This process is typically understood to be goal-directed, helping individuals to meet the demands of their environment and achieve their ambitions (e.g. remaining calm to resolve a conflict) [15]. Whilst the concept of emotion regulation remains unclear by definition, Gross provides a well-defined 'Process model of emotion regulation' [16,17] of specific relevance for research and practice, whereby an individual recognises an emotion regulation goal, selects, and finally implements specific emotion regulation strategies [18]. Gross defined a set of five distinct emotion regulation strategies occurring at different points in an emotional experience: situation selection, situation modification, attentional deployment, cognitive change, and response modulation [18]. Situation selection can be understood as an individual's efforts to alter the likelihood of being in an emotion-evoking situation. Situation modification involves modifying a situation at the time to change its emotional impact. Attentional deployment involves directing attention toward or away from an emotion or its causes. Cognitive change enables reappraising a situation to change its emotional significance. Response modulation includes any efforts to modify the behavioural, experiential, and physiological elements of an emotional response. Each of these strategies can be understood to influence an individual's emotional response in a way that can be interpreted as adaptive (e.g., problem-solving, acceptance) or maladaptive (e.g., withdrawal, suppression) [17], depending on the context.

Maladaptive patterns of emotional experience or expression that interfere with goal-directed activity are typically understood as emotion *dysregulation* and have physiological, cognitive, and social consequences [17,18]. Emotion dysregulation can also be understood to represent problematic emotion dynamics: persistence, lability, and intensity of emotions [19]. Evidence demonstrates emotion dysregulation is present across a range of psychopathologies, including internalising (e.g., generalised anxiety disorder, major depressive disorder, dysthymia) and externalising disorders (e.g., attention-deficit/hyperactivity disorder, conduct disorder, oppositional defiant disorder) [20]. For example, generalised anxiety disorder has been associated with a lack of understanding of emotions and an increased reliance on emotion regulation strategies that could be understood as maladaptive, such as withdrawal [20]. Similarly, attention-deficit/hyperactivity disorder is characterised by individuals' emotion regulation deficits and emotion reactivity [21]. More recent findings have also

For peer review only

For peer review only - http://bmjopen.bmj.com/site/about/guidelines.xhtml

concluded that emotion regulation is a significant risk factor for future mental health difficulties, suggesting it may be a valuable target for treatment and prevention.

Most evidence on the impact of emotion regulation on psychopathology has been derived from adult populations [22]. However, recent findings are beginning to show a close association between emotion regulation and psychopathology in adolescence [23]. Evidence indicates a significant shift in emotion regulation between ages 13 and 15 (e.g., access to strategies, use of adaptive vs. maladaptive strategies), suggesting that adolescence is a particularly vulnerable period in the development of emotion regulation [24]. Therefore, interventions targeting emotion regulation as a transdiagnostic construct central to developing and maintaining psychopathology may reduce the risk and severity of adolescent mental health problems.

Emotion Regulation Interventions

Existing psychological interventions adopt different approaches to improving emotional regulation. Some focus on reducing the use of maladaptive strategies such as rumination (e.g., Rumination-focused cognitive behavioural therapy; RF-CBT [25]), while others focus on increasing the use of adaptive strategies such as acceptance (e.g., Acceptance and Commitment Therapy [26]). The adult literature indicates that such interventions improve emotion regulation and mediate decreases in psychopathological symptoms within certain diagnoses (e.g., anxiety) [27]. However, the effectiveness of these interventions in improving multiple mental health and functional outcomes, rather than specific and individual symptom groups, is largely unknown [28], and the potential utility of emotion dysregulation as a transdiagnostic treatment target for adolescents requires further investigation.

Further, there needs to be more evidence on how these interventions can be rolled out in a sustainable, scalable, and engaging way for adolescents. As the prevalence of adolescent mental health problems increases, innovative solutions must be developed to ensure that a broad range of adolescents can access and engage with support. Much of the research has focused on in-person, one-to-one solutions for emotion regulation and psychopathology [23]. Some innovative self-directed digital interventions targeting emotion dysregulation are beginning to be developed for adolescents [29, 30]. Still, research on their effectiveness in treating or preventing psychopathology is in its infancy.

It is known that digital interventions targeting single conditions have a greater capacity for innovation and engagement with adolescents [31] and the potential to extend effective care cost-effectively and sustainably [32], but it is unclear how digital interventions targeting transdiagnostic mechanisms like emotion regulation can be applied at scale to support this population. Further, more evidence is needed to determine the 'active ingredients' of the interventions that most improve emotion regulation

For peer review only - http://bmjopen.bmj.com/site/about/guidelines.xhtml

and reduce psychopathology in adolescents. For example, emerging evidence suggests that interventions which focus on increasing the use of adaptive emotion regulation strategies rather than reducing the use of maladaptive strategies will be most effective in reducing adolescent mental health problems [33]. As this field emerges, research must assess the differential dimensions of emotion regulation and their impact on psychopathology.

Review Objectives

To address some of these issues, this review will synthesise evidence on current self-directed, digital interventions available to adolescents (aged 11-18 years) and their effectiveness in addressing emotion (dys-)regulation, psychopathology, and functioning (e.g., academic achievement). This review provides an extension to existing work which has thus far summarised the effectiveness of in-person, therapist-supported mental health interventions that target emotion (dys-)regulation for youth (aged 6 – 24 years) [20]. This review takes a more specific focus to develop evidence on a burgeoning number of self-directed, scalable, digital mental health interventions available to adolescents with or without diagnosed psychopathology. Specifically, we will seek to answer the following research questions:

1. Are current self-directed digital interventions that target emotion regulation, effective in improving emotion (dys-)regulation in adolescents?
2. Are these interventions effective in treating psychopathology and improving function (e.g. academic achievement)?
3. What are the specific components (e.g. mood monitoring) of these interventions that most improve emotion (dys-)regulation and/or psychopathology and functioning in adolescents?

METHODS AND ANALYSIS

Protocol and Registration

This protocol is written in accordance with the Preferred Reporting Items for Systematic Review and Meta-Analysis Protocols (PRISMA-P) guidelines [34]. The completed PRISMA-P checklist is provided in *Supplementary file 1*. The systematic review has been registered in PROSPERO, the International Prospective Register of Systematic Reviews (CRD42022385547). Important amendments to this protocol will be reported and published with the review results.

Eligibility Criteria

The following criteria (*Table 1*) are set out to screen identified studies using a PICOS framework [35].

For peer review only

Table 1. *Inclusion and Exclusion Criteria*

	Inclusion	Exclusion
1. Population	• Any adolescent (aged 11-18)<br>• With or without a diagnosed mental health problem<br>• Neurotypical/neurodiverse	• Adolescents outside the specified age range<br>• Non-human participants
2. Intervention	• Any mental health intervention delivered entirely by digital technology (e.g. app-based, web-based etc.)<br>• Can be delivered in any appropriate setting such as at home, in school, in voluntary and community settings, primary, secondary, or tertiary care<br>• Must address emotion (dys-) regulation but this need not be the focus<br>• Must be self-directed	
3. Comparison	• Employing a control group is not an essential criterion for inclusion in the review.	• Employing a control group is not an essential criterion for inclusion in the review.
4. Outcomes	• Must measure emotion (dys-) regulation using a valid or appropriate measure	• If a valid and appropriate item, scale, or measure is not used, the authors of the paper must comment on their reasoning for choosing their measure
5. Study	• Publication dates from 2010 to present<br>• Studies from any geographical location<br>• Primary research study with direct contact or observation of individuals<br>• Grey literature, theses, reports, pre-prints	• Non-English language<br>• Descriptive studies such as case reports/studies that do not include direct contact or observation of individuals<br>• Review papers, books, editorials, commentaries, poster abstracts, conferences

Information Sources

Nine electronic databases will be searched for studies published from 2010: MEDLINE, PsycINFO; Global Health: Scopus; Web of Science Core Collection; EBSCO CINAHL; EBSCO ERIC; OVID Embase; and The Cochrane Central Register of Controlled Trials (CENTRAL). Grey literature such as preprints and theses are also included in this review (databases: HMIC, EThOS, Psyarxiv, Trip,

For peer review only - http://bmjopen.bmj.com/site/about/guidelines.xhtml

ClinicalTrials.gov). This search will be updated periodically to identify any new relevant research from the selected databases. Forward and backward citation tracking from included studies will also be carried out to help identify any other relevant studies.

Search Strategy

The search strategy (*Supplementary File 2*) is designed to identify all studies examining one or more self-directed digital mental health interventions for adolescents, which include an emotion (dys-) regulation component. It will search for synonyms for the following three concepts: *adolescents, emotion (dys-) regulation, and self-directed digital interventions*. The search will be limited to English language studies conducted with a human population.

Study Selection

All identified studies will be exported to Endnote 20, and any duplicates will be removed following a specified method [36] before being imported into Rayyan [37] for further deduplication and screening. Abstracts and titles will be screened based on the inclusion and exclusion criteria (**Table 1**). Those studies that meet these criteria will enter the full-text screening stage for further checks against the eligibility criteria. The full-text screening will allow for the identification of those interventions that do not address emotion (dys-)regulation directly. A second researcher (E.L.) will be assigned a random 10% of the identified studies for screening at the title and abstract stage. Discrepancies throughout the study selection process will be resolved through consensus between AT and EL. If discrepancies cannot be resolved through discussion, a third reviewer will be consulted to adjudicate.

Data Extraction and Management

Data will be extracted and collated by two independent reviewers (A.T. and E.L.) and tracked in Microsoft Excel using a structured coding form and associated coding manual. The form will be piloted on a sample of included studies and refined before extraction begins. Information relating to study characteristics (e.g. author(s), publication date), participant characteristics (e.g. age, gender), digital intervention characteristics (e.g. name, focus), and relevant clinical and emotion dysregulation outcomes will be extracted from each study. Information to determine any study bias will also be collated. Study investigators will be contacted for missing/unreported data or additional details. If we are not able to obtain sufficient details/raw data using this approach, we will exclude the respective studies.

Outcomes

For peer review only - http://bmjopen.bmj.com/site/about/guidelines.xhtml

The primary outcome of this review is the change in emotion (dys-)regulation, psychopathology, and functioning (e.g. academic achievement) occurring as a result of participation in a self-directed digital intervention that addresses emotion (dys-) regulation. Emotion (dys-)regulation must be assessed, and where possible, using a valid and appropriate item, scale, or measure (e.g., Child Social Behaviour Questionnaire) [38], including through clinical interviews or self-reported measures. An existing review of emotion (dys-)regulation assessment [39] and similar reviews [22,40] will be used as guidance to decide on a measure's eligibility. Symptoms of psychopathology will be assessed by any available valid and appropriate measure, including through clinical interview or self-reported measures.

Quality and Risk of Bias Assessment

Two researchers (A.T. and E.L.) will independently assess the methodological quality of the included studies using the Effective Public Health Practice Project Quality Assessment tool (EPHPP). The EPHPP is applicable to a range of quantitative study designs (e.g. case-control studies) and has been judged to be particularly suitable for systematic reviews on the effectiveness of interventions/treatments [41]. Evidence has shown the EPHPP has good content and construct validity [41,42]. The AACODS Checklist will be used to assess the quality and content of any included grey literature (i.e., theses, reports). It has been designed as a critical appraisal tool specifically for use with grey literature sources.

If sufficient data is available, potential reporting bias will be assessed visually using a funnel plot and commented on in the review. An asymmetrical plot suggests a publication bias [43]. If asymmetry in the funnel plot is detected, the studies will be reviewed to assess whether this asymmetry was likely due to publication bias or other factors such as methodological or clinical heterogeneity.

Data Synthesis

Information from the included studies will be synthesised in line with guidance from Popay [44]. A narrative overview of the study characteristics and methodology, participant characteristics and the digital interventions used to address emotion (dys-)regulation will be included. A summary of any observed changes in emotion dysregulation and/or psychopathology occurring as a result of the intervention will be provided. Additionally, any general improvements in functioning (e.g. social, academic) that occur as a result of participation in the intervention will also be described, if reported in the included papers.

Given the findings of preliminary searches and previous reviews [23], it is anticipated that there will be high heterogeneity across studies. However, a meta-analysis will be conducted if the available data

For peer review only - http://bmjopen.bmj.com/site/about/guidelines.xhtml

within the included studies is sufficiently homogenous. Two meta-analyses are planned: one with psychopathology as the primary outcome and one with emotion dysregulation as the primary outcome. Outcomes will be pooled using a random-effects model, in line with current recommendations for meta-analysis models in psychology [45].

Patient and Public Involvement

Since this is a systematic review protocol, no patients or the public were involved.

Ethics and Dissemination

Due to the characteristics of this study design, ethical approval was not required for this study. The findings of this systematic review will be disseminated widely via peer-reviewed publication and presentations at conferences related to this field.

Amendments

Any amendments to this protocol will be documented and reported in the final peer-reviewed publication.

DISCUSSION

This paper summarises the protocol for a systematic review on the effectiveness of self-directed digital interventions in improving emotion (dys-)regulation, psychopathology, and overall functioning in adolescents. The results of this systematic review will establish whether emotion (dys-)regulation is a key transdiagnostic mechanism through which emotion (dys-)regulation-focused, self-directed digital interventions exert their effects across a broad range of important outcomes (i.e. a reduction in symptoms of psychopathology). If effective, these interventions could offer an engaging prevention, early intervention, and treatment approach to improve the quality and reach of mental health support offered to this population.

Previous research with adults has demonstrated that digital interventions focused on emotion (dys-) regulation can improve mental health symptoms such as anxiety, depression, stress, and self-harm. As such, we expect to observe similar improvements in mental health symptoms in adolescents who engage in self-directed digital interventions targeting emotion regulation. If sufficient data is available, we also hope to determine the most important components of these interventions. These findings aim to offer guidance for clinicians and researchers developing or delivering these interventions in practice, improving the quality of support offered to adolescents. This additional outcome has health-economic benefits, ensuring that resources are allocated to those intervention

For peer review only - http://bmjopen.bmj.com/site/about/guidelines.xhtml

components most effective in treating emotion (dys-)regulation and/or psychopathology and functioning in adolescents.

For peer review only

AUTHORS' CONTRIBUTIONS

AT is the guarantor. AT, GH and BO conceived the initial idea for the systematic review. AT drafted the manuscript, and GH and BO provided critical insight. All authors contributed to the revision of the manuscript and approved the final version.

FUNDING STATEMENT

AT and EL are supported by an Economic Social Research Council (ESRC) London Interdisciplinary Social Science Doctoral Training Partnership (LISS-DTP) Postgraduate Scholarship. The funders had no role in study design, data collection and analysis, the decision to publish, or the preparation of the manuscript.

COMPETING INTERESTS STATEMENT

None declared.

For peer review only

REFERENCES

1. Kim-Cohen J, Caspi A, Moffitt TE, *et al*. Prior juvenile diagnoses in adults with mental disorder: developmental follow-back of a prospective-longitudinal cohort. Archives of general psychiatry. 2003 Jul 1;60(7):709-17. doi.org/10.1001/archpsyc.60.7.709

2. Newlove-Delgado, T., Williams, T., Robertson, K., *et al*. Mental Health of Children and Young People in England 2021-wave 2 follow up to the 2017 survey. 2021. Retrieved November 25, 2021, from https://digital.nhs.uk/dataandinformation/publications/statistical/mental-health-of-childrenand-young-people-in-england/2020-wave-1-follow-up.

3. World Health Organization. Adolescent mental health. 2021. Retrieved November 25, 2021, from https://www.who.int/news-room/factsheets/detail/adolescent-mental-health

4. Dalgleish T, Black M, Johnston D, *et al*. Transdiagnostic approaches to mental health problems: Current status and future directions. Journal of consulting and clinical psychology. 2020 Mar;88(3):179. doi.org/10.1037/ccp0000482

5. Uher R, Zwicker A. Etiology in psychiatry: embracing the reality of poly-gene-environmental causation of mental illness. World psychiatry. 2017 Jun;16(2):121-9. doi.org/10.1002/wps.20436

6. Keyes KM, Eaton NR, Krueger RF, *et al*. Childhood maltreatment and the structure of common psychiatric disorders. The British Journal of Psychiatry. 2012 Feb;200(2):107-15. doi.org/10.1192/bjp.bp.111.093062

7. Hosang GM, Johnson SL, Kiecolt-Glaser J, *et al*. Gender specific association of child abuse and adult cardiovascular disease in a sample of patients with Basal Cell Carcinoma. Child Abuse & Neglect. 2013 Jun 1;37(6):374-9. doi.org/10.1016/j.chiabu.2012.09.018

8. Fritz J, De Graaff AM, Caisley H, *et al*. A systematic review of amenable resilience factors that moderate and/or mediate the relationship between childhood adversity and mental health in young people. Frontiers in psychiatry. 2018 Jun 19;9:230. doi.org/10.3389/fpsyt.2018.00230

9. Roefs A, Fried EI, Kindt M, *et al*. A new science of mental disorders: Using personalised, transdiagnostic, dynamical systems to understand, model, diagnose and treat psychopathology. Behaviour Research and Therapy. 2022 Jun 1;153:104096. doi.org/10.1016/j.brat.2022.104096

10. Farchione TJ, Fairholme CP, Ellard KK, *et al*. Unified protocol for transdiagnostic treatment of emotional disorders: a randomized controlled trial. Behavior therapy. 2012 Sep 1;43(3):666-78. doi.org/10.1016/j.beth.2012.01.001

11. Ehrenreich-May J, Rosenfield D, Queen AH, *et al*. An initial waitlist-controlled trial of the unified protocol for the treatment of emotional disorders in adolescents. Journal of Anxiety Disorders. 2017 Mar 1;46:46-55. doi.org/10.1016/j.janxdis.2016.10.006

12. Carlucci L, Saggino A, Balsamo M. On the efficacy of the unified protocol for transdiagnostic treatment of emotional disorders: A systematic review and meta-analysis. Clinical Psychology Review. 2021 Jul 1;87:101999. doi.org/10.1016/j.cpr.2021.101999

13. Forbes MK, Rapee RM, Krueger RF. Opportunities for the prevention of mental disorders by reducing general psychopathology in early childhood. Behaviour research and therapy. 2019 Aug 1;119:103411. doi.org/10.1016/j.brat.2019.103411

14. Aldao A, Gee DG, De Los Reyes A, *et al*. Emotion regulation as a transdiagnostic factor in the development of internalizing and externalizing psychopathology: Current and future directions. Development and psychopathology. 2016 Nov;28(4pt1):927-46. doi.org/10.1017/S0954579416000638

15. D'Agostino A, Covanti S, Rossi Monti M, *et al*. Reconsidering emotion dysregulation. Psychiatric Quarterly. 2017 Dec;88:807-25. doi.org/10.1007/s11126-017-9499-6

For peer review only

For peer review only - http://bmjopen.bmj.com/site/about/guidelines.xhtml

16. Eftekhari A, Zoellner LA, Vigil SA. Patterns of emotion regulation and psychopathology. Anxiety, Stress, & Coping. 2009 Oct 1;22(5):571-86. doi.org/10.1080/10615800802179860

17. Gross JJ. Antecedent-and response-focused emotion regulation: divergent consequences for experience, expression, and physiology. Journal of personality and social psychology. 1998 Jan;74(1):224. doi.org/10.1037/0022-3514.74.1.224

18. Gross JJ. Emotion regulation in adulthood: Timing is everything. Current directions in psychological science. 2001 Dec;10(6):214-9. doi.org/10.1111/1467-8721.00152

19. Thompson RA. Emotion dysregulation: A theme in search of definition. Development and psychopathology. 2019 Aug;31(3):805-15. doi.org/ 10.1017/S0954579419000282

20. Helland SS, Mellblom AV, Kjøbli J, *et al*. Elements in mental health interventions associated with effects on emotion regulation in adolescents: a meta-analysis. Administration and Policy in Mental Health and Mental Health Services Research. 2022 Nov;49(6):1004-18. doi.org/10.1007/s10488-022-01213-2

21. Christiansen H, Hirsch O, Albrecht B, Chavanon ML. Attention-deficit/hyperactivity disorder (ADHD) and emotion regulation over the life span. Current psychiatry reports. 2019 Mar;21:1-1. doi.org/10.1007/s11920-019-1003-6

22. Aldao A, Nolen-Hoeksema S, Schweizer S. Emotion-regulation strategies across psychopathology: A meta-analytic review. Clinical psychology review. 2010 Mar 1;30(2):217-37. doi.org/10.1016/j.cpr.2009.11.004

23. Moltrecht B, Deighton J, Patalay P, *et al*. Effectiveness of current psychological interventions to improve emotion regulation in youth: a meta-analysis. European child & adolescent psychiatry. 2021 Jun;30(6):829-48. doi.org/10.1007/s00787-020-01498-4

24. Zimmermann P, Iwanski A. Emotion regulation from early adolescence to emerging adulthood and middle adulthood: Age differences, gender differences, and emotion-specific developmental variations. International journal of behavioral development. 2014 Mar;38(2):182-94. doi.org/10.1177/0165025413515405

For peer review only

25. Watkins E, Scott J, Wingrove J, *et al*. Rumination-focused cognitive behaviour therapy for residual depression: A case series. Behaviour research and therapy. 2007 Sep 1;45(9):2144-54. doi.org/10.1016/j.brat.2006.09.018

26. Hayes SC, Strosahl KD, Wilson KG. Acceptance and commitment therapy: American Psychological Association. Washington, DC.[Google Scholar]. 2009.

27. Gratz KL, Weiss NH, Tull MT. Examining emotion regulation as an outcome, mechanism, or target of psychological treatments. Current opinion in psychology. 2015 Jun 1;3:85-90. doi.org/10.1016/j.copsyc.2015.02.010

28. Andersen P, Toner P, Bland M, *et al*. Effectiveness of transdiagnostic cognitive behaviour therapy for anxiety and depression in adults: a systematic review and meta-analysis. Behavioural and cognitive psychotherapy. 2016 Nov;44(6):673-90. doi.org/10.1017/S1352465816000229

29. Moltrecht B, Patalay P, Bear HA, *et al*. Interdisciplinary development of a transdiagnostic mobile app to enhance children's emotion regulation: sharing insights and lessons learned. JMIR Formative Research. 2021 Nov 16. doi.org/10.2196/28300

30. Moltrecht B, Patalay P, Deighton J, *et al*. A school-based mobile app intervention for enhancing emotion regulation in children: exploratory trial. JMIR mHealth and uHealth. 2021 Jul 14;9(7):e21837. doi.org/10.2196/21837

31. Liverpool S, Mota CP, Sales CM, *et al*. Engaging children and young people in digital mental health interventions: systematic review of modes of delivery, facilitators, and barriers. Journal of medical Internet research. 2020 Jun 23;22(6):e16317. doi.org/10.2196/16317

32. Mohr DC, Lyon AR, Lattie EG, *et al*. Accelerating digital mental health research from early design and creation to successful implementation and sustainment. Journal of medical Internet research. 2017 May 10;19(5):e7725. doi.org/10.2196/jmir.7725

33. Braet C, Theuwis L, Van Durme K, *et al*. Emotion regulation in children with emotional problems. Cognitive Therapy and Research. 2014 Oct;38:493-504. doi.org/10.1007/s10608-014-9616-x

For peer review only

For peer review only - http://bmjopen.bmj.com/site/about/guidelines.xhtml

34. Moher D, Shamseer L, Clarke M, *et al*, PRISMA-P Group. Preferred reporting items for systematic review and meta-analysis protocols (PRISMA-P) 2015 statement. Syst Rev 2015;4(1):1. doi.org/10.1186/2046-4053-4-1

35. Munn Z, Stern C, Aromataris E, *et al*. What kind of systematic review should I conduct? A proposed typology and guidance for systematic reviewers in the medical and health sciences. BMC medical research methodology. 2018 Dec;18(1):1-9. doi.org/10.1186/s12874-017-0468-4

36. Falconer J. Removing duplicates from an endnote library. [Internet]. . 2018 [cited 2022Apr25]. Available from: https://blogs.lshtm.ac.uk/library/2018/12/07/removing-duplicates-from-an-endnote-library/

37. Ouzzani M, Hammady H, Fedorowicz Z, Elmagarmid A. Rayyan—a web and mobile app for systematic reviews. Systematic reviews. 2016 Dec;5:1-0. doi.org/10.1186/s13643-016-0384-4

38. Luteijn EE, Jackson SA, Volkmar FR, *et al*. The development of the Children's Social Behavior Questionnaire: Preliminary data. Journal of Autism and developmental Disorders. 1998 Dec. doi.org/10.1023/A:1026060330122

39. Adrian M, Zeman J, Erdley C, *et al*. Emotional dysregulation, and interpersonal difficulties as risk factors for nonsuicidal self-injury in adolescent girls. Journal of abnormal child psychology. 2011 Apr;39:389-400. doi.org/10.1007/s10802-010-9465-3

40. Sloan E, Hall K, Moulding R, *et al*. Emotion regulation as a transdiagnostic treatment construct across anxiety, depression, substance, eating and borderline personality disorders: A systematic review. Clinical psychology review. 2017 Nov 1;57:141-63. doi.org/10.1016/j.cpr.2017.09.002

41. Deeks JJ, Dinnes J, D'Amico R, *et al*. Evaluating non-randomised intervention studies. Health technology assessment (Winchester, England). 2003;7(27):iii-173. doi.org/10.3310/hta7270

42. Thomas BH, Ciliska D, Dobbins M, Micucci S. A process for systematically reviewing the literature: providing the research evidence for public health nursing interventions. Worldviews on Evidence-Based Nursing. 2004 Sep;1(3):176-84. doi.org/10.1111/j.1524-475X.2004.04006.x

43. Mavridis D, Salanti G. How to assess publication bias: funnel plot, trim-and-fill method and selection models. BMJ Ment Health. 2014 Feb 1;17(1):30-. doi.org/10.1136/eb-2013-101699

For peer review only - http://bmjopen.bmj.com/site/about/guidelines.xhtml

44. Popay J, Roberts H, Sowden A, *et al*. Guidance on the conduct of narrative synthesis in systematic reviews. A product from the ESRC methods programme Version. 2006 Apr 1;1(1):b92.

45. Schmidt FL, Oh IS, Hayes TL. Fixed-versus random-effects models in meta-analysis: Model properties and an empirical comparison of differences in results. British Journal of Mathematical and Statistical Psychology. 2009 Feb;62(1):97-128. doi.org/10.1348/000711007X255327

For peer review only

For peer review only - http://bmjopen.bmj.com/site/about/guidelines.xhtml

Database: Ovid PsycINFO 1806 to present
Search Strategy:

----

1 (adolescen* or juvenil* or youth* or teen* or pubescen* or pediatric* or paediatric* or "high school" or "secondary school" or school* or "young people" or "young person*" or "young adult*" or "young man" or "young men" or "young woman" or "young women" or student*).ti,ab

2 (emotion* AND (regulat* or dysregulat* or generat* OR modulat* OR adapt* OR cop* OR react* OR arous* OR avoid* OR behavio* OR suppress* OR ruminat*)).,ti,ab

3 (affect AND (regulat* or dysregulat* or generat* OR modulat* OR adapt* OR cop* OR react* OR arous* OR avoid* OR behavio* OR suppress* OR ruminat*)).ti,ab

4 ("self-concept" OR "mood regulat*" OR "situation selection" OR "situation modification" OR "attentional deployment" OR "response modulation" OR "problem focused coping" OR "impulsivity" OR "hyper-reactivity" OR "anger regulation" OR "experiential avoidance" OR "expressive suppression" OR "thought suppression" OR "ruminat*" OR "emotion regulation questionnaire" OR "Difficulties in Emotion Regulation Scale" OR "emotion regulation strategy").ti,ab

5 2 or 3 or 4

6 (treat* or interven* or "early intervention" or "self-directed intervention" or "self-directed" or "public health").ti,ab.

7 phon* or mobile* or "smart-phone*" or device* or computer* or tablet* or "device-based" or "app-based" or apps or app or "sensor*" or "social media").ti,ab.

8 ((digital or virtual or online or interactive) and (health* or intervention or support or platform*)).ti,ab.

9 ("eHealth" or "e-health" or "m-health" or mHealth or "tele-health" or telehealth).ti,ab.

10 7 or 8 or 9

11 1 and 5 and 6 and 10

12 limit 11 to (peer reviewed journal and human and english language and (childhood <birth to 12 years> or adolescence <13 to 17 years>) and ("0100 journal" or "0110 peer-reviewed journal") and journal article and english and human and yr="1995 -Current")

For peer review only

Database: PubMed MEDLINE

Search Strategy:

----

1 adolescen*[Title/Abstract] OR juvenil*[Title/Abstract] OR youth*[Title/Abstract] OR teen*[Title/Abstract] OR pubescen*[Title/Abstract] OR pediatric*[Title/Abstract] OR paediatric*[Title/Abstract] OR "high school"[Title/Abstract] OR "secondary school"[Title/Abstract] OR school*[Title/Abstract] OR "young people"[Title/Abstract] OR "young person*"[Title/Abstract] OR "young adult*"[Title/Abstract] OR "young man"[Title/Abstract] OR "young men"[Title/Abstract] OR "young woman"[Title/Abstract] OR "young women"[Title/Abstract] OR student*[Title/Abstract]

2 emotion*[Title/Abstract] AND (regulat*[Title/Abstract] OR dysregulat*[Title/Abstract] OR generat*[Title/Abstract] OR modulat*[Title/Abstract] OR adapt*[Title/Abstract] OR react*[Title/Abstract] OR arous*[Title/Abstract] OR avoid*[Title/Abstract] OR behavio*[Title/Abstract] OR suppress*[Title/Abstract] OR ruminat*[Title/Abstract])

3 affect[Title/Abstract] AND (regulat*[Title/Abstract] OR dysregulat*[Title/Abstract] OR generat*[Title/Abstract] OR modulat*[Title/Abstract] OR adapt*[Title/Abstract] OR react*[Title/Abstract] OR arous*[Title/Abstract] OR avoid*[Title/Abstract] OR behavio*[Title/Abstract] OR suppress*[Title/Abstract] OR ruminat*[Title/Abstract])

4 "self-concept"[Title/Abstract] OR "mood regulat*"[Title/Abstract] OR "situation selection"[Title/Abstract] OR "situation modification"[Title/Abstract] OR "attentional deployment"[Title/Abstract] OR "response modulation"[Title/Abstract] OR "problem focused coping"[Title/Abstract] OR "impulsivity"[Title/Abstract] OR "hyper-reactivity"[Title/Abstract] OR "anger regulation"[Title/Abstract] OR "experiential avoidance"[Title/Abstract] OR "expressive suppression"[Title/Abstract] OR "thought suppression"[Title/Abstract] OR "ruminat*"[Title/Abstract] OR "emotion regulation questionnaire"[Title/Abstract] OR "Difficulties in Emotion Regulation Scale"[Title/Abstract] OR "emotion regulation strategy"[Title/Abstract]

5 #2 OR #3 OR #4

6 treat*[Title/Abstract] OR interven*[Title/Abstract] OR "early intervention"[Title/Abstract] OR "self-directed intervention"[Title/Abstract] OR "self-directed"[Title/Abstract] OR "public health"[Title/Abstract]

7 phon*[Title/Abstract] OR mobile*[Title/Abstract] OR "smart-phone*"[Title/Abstract] OR device*[Title/Abstract] OR computer*[Title/Abstract] OR tablet*[Title/Abstract] OR "device-based"[Title/Abstract] OR "app-based"[Title/Abstract] OR apps[Title/Abstract] OR app[Title/Abstract] OR "sensor*"[Title/Abstract] OR "social media"[Title/Abstract]

8 ((digital[Title/Abstract] OR virtual[Title/Abstract] OR online[Title/Abstract] OR interactive[Title/Abstract]) AND (health*[Title/Abstract] OR intervention[Title/Abstract] OR support[Title/Abstract] OR platform*[Title/Abstract]))

For peer review only

9 "eHealth"[Title/Abstract] OR "e-health"[Title/Abstract] OR "m-health"[Title/Abstract] OR mHealth[Title/Abstract] OR "tele-health"[Title/Abstract] OR telehealth[Title/Abstract]

10 #7 OR #8 OR #9

11 #1 and #5 and #6 and #10

12 #1 and #5 and #6 and #10 Filters: Humans, English, MEDLINE, from 1995 – 2023

For peer review only

Database: Scopus

Search Strategy:

(TITLE-ABS-KEY (adolescen* OR juvenil* OR youth* OR teen* OR pubescen* OR pediatric* OR paediatric* OR "high school" OR "secondary school" OR school* OR "young people" OR "young person*" OR "young adult*" OR "young man" OR "young men" OR "young woman" OR "young women" OR student*) AND TITLE-ABS-KEY ((emotion* AND (regulat* OR dysregulat* OR generat* OR modulat* OR adapt* OR cop* OR react* OR arous* OR avoid* OR behavio* OR suppress* OR ruminat*)) OR (affect AND (regulat* OR dysregulat* OR generat* OR modulat* OR adapt* OR cop* OR react* OR arous* OR avoid* OR behavio* OR suppress* OR ruminat*)) OR (self-concept OR "mood regulat*" OR "situation selection" OR "situation modification" OR "attentional deployment" OR "response modulation" OR "problem focused coping" OR impulsivity OR hyper-reactivity OR "anger regulation" OR "experiential avoidance" OR "expressive suppression" OR "thought suppression" OR ruminat* OR "emotion regulation questionnaire" OR "Difficulties in Emotion Regulation Scale" OR "emotion regulation strategy")) AND TITLE-ABS-KEY (treat* OR interven* OR "early intervention" OR "self-directed intervention" OR "self-directed" OR "public health") AND TITLE-ABS-KEY ((phon* OR mobile* OR "smart-phone*" OR device* OR computer* OR tablet* OR "device-based" OR "app-based" OR apps OR app OR "sensor*" OR "social media") OR ((digital OR virtual OR online OR interactive) AND (health* OR intervention OR support OR platform*)) OR ("eHealth" OR "e-health" OR "m-health" OR mhealth OR "tele-health" OR telehealth))) AND ORIG-LOAD-DATE AFT 20230203 AND (LIMIT-TO (SUBJAREA , "MEDI") OR LIMIT-TO (SUBJAREA , "SOCI") OR LIMIT-TO (SUBJAREA , "MULT") OR LIMIT-TO (SUBJAREA , "PSYC") OR LIMIT-TO (SUBJAREA , "NURS") OR LIMIT-TO (SUBJAREA , "HEAL")) AND (LIMIT-TO (DOCTYPE , "ar")) AND (LIMIT-TO (EXACTKEYWORD , "Human") OR LIMIT-TO (EXACTKEYWORD , "Humans") OR LIMIT-TO (EXACTKEYWORD , "Adolescent")) AND (LIMIT-TO (LANGUAGE , "English")) AND (EXCLUDE (SUBJAREA , "MEDI") OR EXCLUDE (SUBJAREA , "NEUR") OR EXCLUDE (SUBJAREA , "BIOC") OR EXCLUDE (SUBJAREA , "ARTS") OR EXCLUDE (SUBJAREA , "ENVI") OR EXCLUDE (SUBJAREA , "PHAR")) AND (EXCLUDE (SUBJAREA , "AGRI") OR EXCLUDE (SUBJAREA , "BUSI") OR EXCLUDE (SUBJAREA , "COMP") OR EXCLUDE (SUBJAREA , "DENT") OR EXCLUDE (SUBJAREA , "ENGI")) AND (EXCLUDE (SUBJAREA , "VETE"))

For peer review only

Database: Global Health 1973 to 2022 Week 50

Search Strategy:

----

1 (adolescen* or juvenil* or youth* or teen* or pubescen* or pediatric* or paediatric* or "high school" or "secondary school" or school* or "young people" or "young person*" or "young adult*" or "young man" or "young men" or "young woman" or "young women" or student*).ti,ab

2 (emotion* AND (regulat* or dysregulat* or generat* OR modulat* OR adapt* OR cop* OR react* OR arous* OR avoid* OR behavio* OR suppress* OR ruminat*)).,ti,ab

3 (affect AND (regulat* or dysregulat* or generat* OR modulat* OR adapt* OR cop* OR react* OR arous* OR avoid* OR behavio* OR suppress* OR ruminat*)).,ti,ab

4 ("self-concept" OR "mood regulat*" OR "situation selection" OR "situation modification" OR "attentional deployment" OR "response modulation" OR "problem focused coping" OR "impulsivity" OR "hyper-reactivity" OR "anger regulation" OR "experiential avoidance" OR "expressive suppression" OR "thought suppression" OR "ruminat*" OR "emotion regulation questionnaire" OR "Difficulties in Emotion Regulation Scale" OR "emotion regulation strategy").ti,ab

5 2 or 3 or 4

6 (treat* or interven* or "early intervention" or "self-directed intervention" or "self-directed" or "public health").ti,ab.

7 phon* or mobile* or "smart-phone*" or device* or computer* or tablet* or "device-based" or "app-based" or apps or app or "sensor*" or "social media").ti,ab.

8 ((digital or virtual or online or interactive) and (health* or intervention or support or platform*)).ti,ab.

9 ("eHealth" or "e-health" or "m-health" or mHealth or "tele-health" or telehealth).ti,ab.

10 7 or 8 or 9

11 1 and 5 and 6 and 10

12 limit 11 to (english language and english and (journal or journal article) and yr="1995 -Current")

For peer review only

Database: EBSCO CINAHL

Search Strategy:

----

1 AB adolescen* or juvenil* or youth* or teen* or pubescen* or pediatric* or paediatric* or "high school" or "secondary school" or school* or "young people" or "young person*" or "young adult*" or "young man" or "young men" or "young woman" or "young women" or student*

2 AB emotion* AND (regulat* or dysregulat* or generat* OR modulat* OR adapt* OR cop* OR react* OR arous* OR avoid* OR behavio* OR suppress* OR ruminat*)

3 AB affect AND (regulat* or dysregulat* or generat* OR modulat* OR adapt* OR cop* OR react* OR arous* OR avoid* OR behavio* OR suppress* OR ruminat*)

4 AB "self-concept" OR "mood regulat*" OR "situation selection" OR "situation modification" OR "attentional deployment" OR "response modulation" OR "problem focused coping" OR "impulsivity" OR "hyper-reactivity" OR "anger regulation" OR "experiential avoidance" OR "expressive suppression" OR "thought suppression" OR "ruminat*" OR "emotion regulation questionnaire" OR "Difficulties in Emotion Regulation Scale" OR "emotion regulation strategy"

5 S2 or S3 or S4

6 AB treat* or interven* or "early intervention" or "self-directed intervention" or "self-directed" or "public health"

7 AB phon* or mobile* or "smart-phone*" or device* or computer* or tablet* or "device-based" or "app-based" or apps or app or "sensor*" or "social media"

8 AB (digital or virtual or online or interactive) and (health* or intervention or support or platform*))

9 AB "eHealth" or "e-health" or "m-health" or mHealth or "tele-health" or telehealth

10 S7 or S8 or S9

11 S1 and S5 and S6 and S10

Limiters - Published Date: 19950101-; English Language; Peer Reviewed; Research Article; Human; Publication Type: Journal Article; Age Groups: Child: 6-12 years, Adolescent: 13-18 years; Language: English

For peer review only

Database: EBSCO ERIC

Search Strategy:

----

1 AB adolescen* or juvenil* or youth* or teen* or pubescen* or pediatric* or paediatric* or "high school" or "secondary school" or school* or "young people" or "young person*" or "young adult*" or "young man" or "young men" or "young woman" or "young women" or student*

2 AB emotion* AND (regulat* or dysregulat* or generat* OR modulat* OR adapt* OR cop* OR react* OR arous* OR avoid* OR behavio* OR suppress* OR ruminat*)

3 AB affect AND (regulat* or dysregulat* or generat* OR modulat* OR adapt* OR cop* OR react* OR arous* OR avoid* OR behavio* OR suppress* OR ruminat*)

4 AB "self-concept" OR "mood regulat*" OR "situation selection" OR "situation modification" OR "attentional deployment" OR "response modulation" OR "problem focused coping" OR "impulsivity" OR "hyper-reactivity" OR "anger regulation" OR "experiential avoidance" OR "expressive suppression" OR "thought suppression" OR "ruminat*" OR "emotion regulation questionnaire" OR "Difficulties in Emotion Regulation Scale" OR "emotion regulation strategy"

5 S2 or S3 or S4

6 AB treat* or interven* or "early intervention" or "self-directed intervention" or "self-directed" or "public health"

7 AB phon* or mobile* or "smart-phone*" or device* or computer* or tablet* or "device-based" or "app-based" or apps or app or "sensor*" or "social media"

8 AB (digital or virtual or online or interactive) and (health* or intervention or support or platform*)

9 AB "eHealth" or "e-health" or "m-health" or mHealth or "tele-health" or telehealth

10 S7 or S8 or S9

11 S1 and S5 and S6 and S10

Limiters - Published Date: 19950101-; English Language; Peer Reviewed; Research Article; Human; Publication Type: Journal Article; Age Groups: Child: 6-12 years, Adolescent: 13-18 years; Language: English

For peer review only

Database: The Cochrane Central Register of Controlled Trials (CENTRAL)
Search Strategy:

----

1 adolescen* or juvenil* or youth* or teen* or pubescen* or pediatric* or paediatric* or "high school" or "secondary school" or school* or "young people" or "young person*" or "young adult*" or "young man" or "young men" or "young woman" or "young women" or student*

2 emotion* AND (regulat* or dysregulat* or generat* OR modulat* OR adapt* OR cop* OR react* OR arous* OR avoid* OR behavio* OR suppress* OR ruminat*)

3 affect AND (regulat* or dysregulat* or generat* OR modulat* OR adapt* OR cop* OR react* OR arous* OR avoid* OR behavio* OR suppress* OR ruminat*)

4 self-concept OR "mood regulat*" OR "situation selection" OR "situation modification" OR "attentional deployment" OR "response modulation" OR "problem focused coping" OR "impulsivity" OR hyper-reactivity OR "anger regulation" OR "experiential avoidance" OR "expressive suppression" OR "thought suppression" OR ruminat* OR "emotion regulation questionnaire" OR "Difficulties in Emotion Regulation Scale" OR "emotion regulation strategy"

5 #2 or #3 or #4

6 treat* or interven* or "early intervention" or "self-directed intervention" or "self-directed" or "public health"

7 phon* or mobile* or "smart-phone*" or device* or computer* or tablet* or "device-based" or "app-based" or apps or app or "sensor*" or "social media"

8 (digital or virtual or online or interactive) and (health* or intervention or support or platform*)

9 "eHealth" or "e-health" or "m-health" or mHealth or "tele-health" or telehealth

10 #7 or #8 or #9

11 #1 and #5 and #6 and #10

with Publication Year from 1995 to 2023, in Trials with Public Health, Developmental, Psychosocial and Learning Problems, Child Health in Cochrane Groups

For peer review only

Database: Web of Science: Core Collection
Search Strategy:

----

1 AB=(adolescen* or juvenil* or youth* or teen* or pubescen* or pediatric* or paediatric* or "high school" or "secondary school" or school* or "young people" or "young person*" or "young adult*" or "young man" or "young men" or "young woman" or "young women" or student*)
2 AB=(emotion* AND (regulat* or dysregulat* or generat* OR modulat* OR adapt* OR cop* OR react* OR arous* OR avoid* OR behavio* OR suppress* OR ruminat*))
3 AB=(affect* AND (regulat* or dysregulat* or generat* OR modulat* OR adapt* OR cop* OR react* OR arous* OR avoid* OR behavio* OR suppress* OR ruminat*))
4 AB=(self-concept OR "mood regulat*" OR "situation selection" OR "situation modification" OR "attentional deployment" OR "response modulation" OR "problem focused coping" OR "impulsivity" OR hyper-reactivity OR "anger regulation" OR "experiential avoidance" OR "expressive suppression" OR "thought suppression" OR ruminat* OR "emotion regulation questionnaire" OR "Difficulties in Emotion Regulation Scale" OR "emotion regulation strategy")
5 #2 OR #3 OR #4

6 AB=(treat* or interven* or "early intervention" or "self-directed intervention" or "self-directed" or "public health")

7 AB=(phon* or mobile* or "smart-phone*" or device* or computer* or tablet* or "device-based" or "app-based" or apps or app or "sensor*" or "social media")

8 AB=((digital or virtual or online or interactive) and (health* or intervention or support or platform*))

9 AB=("eHealth" or "e-health" or "m-health" or mHealth or "tele-health" or telehealth)

10 #9 OR #8 OR #7

11 #10 AND #6 AND #5 AND #1

18 #17 and 2023 or 2022 or 2021 or 2020 or 2019 or 2000 or 2001 or 2002 or 2003 or 2004 or 2005 or 2006 or 2007 or 2008 or 2009 or 2010 or 2011 or 2012 or 2013 or 2014 or 2015 or 2016 or 2017 or 2018 (Publication Years) and English (Languages) and English (Languages) and Article (Document Types)

19 #10 AND #6 AND #5 AND #1 and 2023 or 2022 or 2021 or 2020 or 2019 or 2018 or 2017 or 2016 or 2015 or 2014 or 2013 or 2012 or 2011 or 2001 or 2002 or 2003 or 2004 or 2006 or 2007 or 2009 or 2010 or 2005 or 2008 or 1995 or 1996 or 1997 or 1998 or 1999 or 2000 (Publication Years) and Psychiatry or Public Environmental Occupational Health or Education Educational Research or Psychology Multidisciplinary or Psychology Clinical or Pediatrics or Health Care Sciences Services or Psychology Developmental or Psychology or Family Studies or Multidisciplinary Sciences or Nursing or Medicine Research Experimental or Medicine General Internal or Psychology Educational or Behavioral Sciences or Education Scientific Disciplines or Health Policy Services or Social Sciences Interdisciplinary or Social Work or Education Special or Psychology Social or Psychology Applied or Psychology Experimental or Sociology (Web of Science Categories) and

For peer review only

Book Review or Data Paper or Reprint or Editorial Material or Book Chapters or Biographical-Item or Review Article or Proceeding Paper or Early Access (Exclude – Document Types) and English (Languages) and Psychology or Psychiatry or Public Environmental Occupational Health or Health Care Sciences Services or Education Educational Research or Pediatrics or General Internal Medicine or Nursing or Family Studies or Science Technology Other Topics or Research Experimental Medicine or Behavioral Sciences or Social Sciences Other Topics or Social Work (Research Areas)

For peer review only

Database: Elsevier Embase 1947 to present

Search Strategy:

----

1 (adolescen* or juvenil* or youth* or teen* or pubescen* or pediatric* or paediatric* or "high school" or "secondary school" or school* or "young people" or "young person*" or "young adult*" or "young man" or "young men" or "young woman" or "young women" or student*).ti,ab

2 (emotion* AND (regulat* or dysregulat* or generat* OR modulat* OR adapt* OR cop* OR react* OR arous* OR avoid* OR behavio* OR suppress* OR ruminat*)).,ti,ab

3 (affect AND (regulat* or dysregulat* or generat* OR modulat* OR adapt* OR cop* OR react* OR arous* OR avoid* OR behavio* OR suppress* OR ruminat*)).,ti,ab

4 ("self-concept" OR "mood regulat*" OR "situation selection" OR "situation modification" OR "attentional deployment" OR "response modulation" OR "problem focused coping" OR "impulsivity" OR "hyper-reactivity" OR "anger regulation" OR "experiential avoidance" OR "expressive suppression" OR "thought suppression" OR "ruminat*" OR "emotion regulation questionnaire" OR "Difficulties in Emotion Regulation Scale" OR "emotion regulation strategy").ti,ab

5 2 or 3 or 4

6 (treat* or interven* or "early intervention" or "self-directed intervention" or "self-directed" or "public health").ti,ab.

7 phon* or mobile* or "smart-phone*" or device* or computer* or tablet* or "device-based" or "app-based" or apps or app or "sensor*" or "social media").ti,ab.

8 ((digital or virtual or online or interactive) and (health* or intervention or support or platform*)).ti,ab.

9 ("eHealth" or "e-health" or "m-health" or mHealth or "tele-health" or telehealth).ti,ab.

10 7 or 8 or 9

11 1 and 5 and 6 and 10

12 limit 11 to (human and english language and "remove medline records" and embase and yr="1995 -Current" and journal)

For peer review only

Database: EThOS (PhD Theses)

Search Strategy:

1	"adolescen\" OR "high school" OR "secondary school" OR "youth" OR "young person" OR "young people"	Any word
2	AND "emotional regulation" OR "mood regulat\"	Any word
3	OR "emotion\" AND ("dysregulat\" OR "generat\" OR "modulat\" OR "adapt\" OR "cop\" OR react\ OR arous\ OR avoid\ OR behavio\ OR suppress\" OR "ruminat\")	Any word
4	OR affect AND (regulat\ or dysregulat\ or generat\ OR modulat\ OR adapt\ OR cop\ OR react\ OR arous\ OR avoid\ OR behavio\ OR "suppress\" OR "ruminat\”)	Any word
5	AND "prevent\" OR "promot\" OR "treat\" OR "evaluat\" OR "effective\" OR "interven\" OR "early intervention" OR "public health" OR "therap\"	Any word
6	AND "telemedicine\" OR "e-health\" OR "app-based" OR "virtual\" OR "web-based" OR "online\" OR "wearable" OR "device" OR "digital" OR "remote"	Any word

Database: OATD (PhD Theses)

Search Strategy:

1	"adolescen*" OR "youth*" OR "high school" OR "secondary school" OR "young person" OR "young people"	Any word
2	AND ("emotional regulation" OR "mood regulat*") OR ("emotion*" AND ("dysregulat*" OR "generat*" OR "modulat*" OR "adapt*" OR "cop*" OR react* OR arous* OR avoid* OR behavio* OR suppress*" OR "ruminat*")) OR (affect AND (regulat* or dysregulat* or generat* OR modulat* OR adapt* OR cop* OR react* OR arous* OR avoid* OR behavio* OR "suppress*" OR "ruminat*”))	Any word
3	AND "prevent*" OR "promot*" OR "treat*" OR "evaluat*" OR "effective*" OR "interven*" OR "early intervention" OR "public health" OR "therap*"	Any word
4	AND "telemedicine/" OR "e-health/" OR "app-based" OR "virtual*" OR "web-based" OR "online*" OR "wearable" OR "device" OR "digital" OR "remote"	Any word

For peer review only

Database: HMIC Health Management Information Consortium *1979 to November 2022*
Search Strategy:
----

1 exp adolescents/ or adolescent development/ or young adults/ or youth/
2 (adolescen* or juvenil* or youth* or teen* or pubescen* or pediatric* or paediatric* or "high school" or "secondary school" or school* or "young people" or "young person*" or "young adult*" or "young man" or "young men" or "young woman" or "young women" or student*).ti,ab.
3 1 or 2
4 "emotional regulation".mp.
5 (emotion* AND (regulat* or dysregulat* or generat* OR modulat* OR adapt* OR cop* OR react* OR arous* OR avoid* OR behavio* OR suppress* OR ruminat*)).ti,ab.
6 (affect AND (regulat* or dysregulat* or generat* OR modulat* OR adapt* OR cop* OR react* OR arous* OR avoid* OR behavio* OR suppress* OR ruminat*)).ti,ab
7 ("self-concept" OR "mood regulat*" OR "situation selection" OR "situation modification" OR "attentional deployment" OR "response modulation" OR "problem focused coping" OR impulsivity OR "hyper-reactivity" OR "anger regulation" OR "experiential avoidance" OR "behavio* avoidance" OR "expressive suppression" OR "thought suppression" OR ruminat* OR "emotion regulation questionnaire" OR "Difficulties in Emotion Regulation Scale" OR "emotion regulation strategy").ti,ab.
8 4 or 5 or 6 or 7
9 (prevent* or promot* or treat* or evaluat* or effective*).ti,ab.
10 (interven* or "early intervention" or "public health" or therap*).ti,ab.
11 9 or 10
12 telemedicine/ or e-health/
13 (phon* or mobile* or "smart-phone*" or device* or computer* or tablet* or "device-based" or "app-based" or apps or app or "sensor*" or "smart-home*" or "social media").ti,ab.
14 (tele and (phone* or nursing or health* or medicine or intervention or informatics or care or support or monitor* or presence or consultat* or rehab* or communicat*)).ti,ab.
15 (digital and (nursing* or health* or medicine or intervention or informatics or care or support or monitor* or presence or consultat* or rehab* or communicat* or platform*)).ti,ab.
16 (virtual and (nursing* or health* or medicine or intervention or informatics or care or support or monitor* or presence or consultat* or rehab* or communicat* or reality* or platform*)).ti,ab.
17 (online and (nursing* or health* or medicine or intervention or informatics or care or support or monitor* or presence or consultat* or rehab* or communicat* or platform*)).ti,ab

For peer review only

18	(interactiv* or "eHealth" or "e-health" or "e-therapy" or "e-learn" or "m-health").ti,ab.
19	("wireless health" or "health telematics" or "healthcare technology" or "therapeutic technology" or online or "medical informatics" or "medical information system" or "electronic health information" or "mobile application*" or "web-based" or website or wifi or "internet-delivered" or "internet-based" or "smart toy" OR "wearable*").ti,ab.
20	12 or 13 or 14 or 15 or 16 or 17 or 18 or 19
21	3 and 8 and 11 and 20
**22**	**limit 21 to (English language and yr="2000-2022")**

For peer review only

Database: PsyArXiv
Search Strategy:

("emotional regulation" OR "mood regulat*" OR "emotion dysregulat*" OR "arous*" OR "avoid*" OR "behavio*" OR "suppress*" OR "ruminat*") AND ("prevent*" OR "promot*" OR "treat*" OR "evaluat*" OR "effective*" OR "interven*" OR "early intervention" OR "public health" OR "therap*") AND ("telemedicine" OR "e-health" OR "app-based" OR "virtual*" OR "web-based" OR "online*" OR "wearable" OR "device" OR "digital" OR "remote")

For peer review only

Database: TRIP

Search Strategy:

("adolescen*" OR "youth*" OR "high school" OR "secondary school" OR "young person" OR "young people") AND ("emotional regulation" OR "mood regulat*" OR "emotion dysregulat*" OR "arous*" OR "avoid*" OR "behavio*" OR "suppress*" OR "ruminat*") AND ("prevent*" OR "promot*" OR "treat*" OR "evaluat*" OR "effective*" OR "interven*" OR "early intervention" OR "public health" OR "therap*") AND ("telemedicine" OR "e-health" OR "app-based" OR "virtual*" OR "web-based" OR "online*" OR "wearable" OR "device" OR "digital" OR "remote")

For peer review only

Database: ClinicalTrials.gov

Search Strategy:

("telemedicine" OR "e-health" OR "virtual*" OR "digital") | Emotional Regulation | Child

For peer review only

PRISMA-P (Preferred Reporting Items for Systematic review and Meta-Analysis Protocols) 2015 checklist: recommended items to address in a systematic review protocol*

Section and topic		Item No	Checklist item
**ADMINISTRATIVE INFORMATION**			
Title:			
Identification	✓	1a	Identify the report as a protocol of a systematic review
Update	N/A	1b	If the protocol is for an update of a previous systematic review, identify as such
Registration	✓	2	If registered, provide the name of the registry (such as PROSPERO) and registration number
Authors:			
Contact	✓	3a	Provide name, institutional affiliation, e-mail address of all protocol authors; provide physical mailing address of corresponding author
Contributions	✓	3b	Describe contributions of protocol authors and identify the guarantor of the review
Amendments	✓	4	If the protocol represents an amendment of a previously completed or published protocol, identify as such and list changes; otherwise, state plan for documenting important protocol amendments
Support:			
Sources	✓	5a	Indicate sources of financial or other support for the review
Sponsor	✓	5b	Provide name for the review funder and/or sponsor
Role of sponsor or funder	✓	5c	Describe roles of funder(s), sponsor(s), and/or institution(s), if any, in developing the protocol
**INTRODUCTION**			
Rationale	✓	6	Describe the rationale for the review in the context of what is already known
Objectives	✓	7	Provide an explicit statement of the question(s) the review will address with reference to participants, interventions, comparators, and outcomes (PICO)
**METHODS**			
Eligibility criteria	✓	8	Specify the study characteristics (such as PICO, study design, setting, time frame) and report characteristics (such as years considered, language, publication status) to be used as criteria for eligibility for the review
Information sources	✓	9	Describe all intended information sources (such as electronic databases, contact with study authors, trial registers or other grey literature sources) with planned dates of coverage
Search strategy	✓	10	Present draft of search strategy to be used for at least one electronic database, including planned limits, such that it could be repeated
Study records:			
Data management	✓	11a	Describe the mechanism(s) that will be used to manage records and data throughout the review

For peer review only

Selection process	✓	11b	State the process that will be used for selecting studies (such as two independent reviewers) through each phase of the review (that is, screening, eligibility and inclusion in meta-analysis)
Data collection process	✓	11c	Describe planned method of extracting data from reports (such as piloting forms, done independently, in duplicate), any processes for obtaining and confirming data from investigators
Data items	✓	12	List and define all variables for which data will be sought (such as PICO items, funding sources), any pre-planned data assumptions and simplifications
Outcomes and prioritization	✓	13	List and define all outcomes for which data will be sought, including prioritization of main and additional outcomes, with rationale
Risk of bias in individual studies	✓	14	Describe anticipated methods for assessing risk of bias of individual studies, including whether this will be done at the outcome or study level, or both; state how this information will be used in data synthesis
Data synthesis	✓	15a	Describe criteria under which study data will be quantitatively synthesised
	✓	15b	If data are appropriate for quantitative synthesis, describe planned summary measures, methods of handling data and methods of combining data from studies, including any planned exploration of consistency (such as I^2, Kendall's τ)
	✓	15c	Describe any proposed additional analyses (such as sensitivity or subgroup analyses, meta-regression)
	✓	15d	If quantitative synthesis is not appropriate, describe the type of summary planned
Meta-bias(es)	✓	16	Specify any planned assessment of meta-bias(es) (such as publication bias across studies, selective reporting within studies)
Confidence in cumulative evidence	✓	17	Describe how the strength of the body of evidence will be assessed (such as GRADE)

*** It is strongly recommended that this checklist be read in conjunction with the PRISMA-P Explanation and Elaboration (cite when available) for important clarification on the items. Amendments to a review protocol should be tracked and dated. The copyright for PRISMA-P (including checklist) is held by the PRISMA-P Group and is distributed under a Creative Commons Attribution Licence 4.0.**

From: Shamseer L, Moher D, Clarke M, Ghersi D, Liberati A, Petticrew M, Shekelle P, Stewart L, PRISMA-P Group. Preferred reporting items for systematic review and meta-analysis protocols (PRISMA-P) 2015: elaboration and explanation. BMJ. 2015 Jan 2;349(jan02 1):g7647.

For peer review only

BMJ Open

Self-Directed Digital Interventions for the Improvement of Emotion Regulation - Effectiveness for Mental Health and Functioning in Adolescents: Protocol for a Systematic Review

Journal:	*BMJ Open*
Manuscript ID	bmjopen-2023-081556.R1
Article Type:	Protocol
Date Submitted by the Author:	22-Feb-2024
Complete List of Authors:	Thomson, Abigail; Queen Mary University of London, Centre for Psychiatry and Mental Health, Wolfson Institute of Population Health<br>Lawrence, Erin; Queen Mary University of London, Centre for Psychiatry and Mental Health, Wolfson Institute of Population Health<br>Oliver, Bonamy; University College London, UCL Institute of Education<br>Wright, Ben; East London NHS Foundation Trust; City University of London<br>Hosang, Georgina M.; Queen Mary University of London, Centre for Psychiatry and Mental Health, Wolfson Institute of Population Health
**Primary Subject Heading**:	Mental health
Secondary Subject Heading:	Evidence based practice
Keywords:	Adolescents < Adolescent, MENTAL HEALTH, eHealth

SCHOLARONE™
Manuscripts

Review Protocol

Title: Self-Directed Digital Interventions for the Improvement of Emotion Regulation - Effectiveness for Mental Health and Functioning in Adolescents: Protocol for a Systematic Review

ABSTRACT

Introduction

Research suggests that problems with emotion regulation, that is, how a person manages and responds to an emotional experience, are related to a range of psychological disorders (e.g. bipolar disorder, anxiety, and depression). Interventions targeting emotion regulation have been shown to improve mental health in adults, but evidence on related interventions for adolescents is still emerging. Increasingly, self-directed digital interventions (e.g. mobile apps) are being developed to target emotion regulation in this population, but questions remain about their effectiveness. This systematic review aimed to synthesise evidence on current self-directed digital interventions available to adolescents (aged 11-18 years) and their effectiveness in addressing emotion regulation, psychopathology, and functioning (e.g., academic achievement).

Methods and Analysis

Several electronic databases will be searched (e.g. MEDLINE, PsycINFO, ACM Digital Library) to identify all studies published any time after January 2010 examining self-directed digital interventions for adolescents, which include an emotion regulation component. This search will be updated periodically to identify any new relevant research from the selected databases. Data on the study characteristics (e.g. author(s)) and methodology, participant characteristics (e.g. age) and the digital interventions used to address emotion (dys-)regulation (e.g. name, focus) will be extracted. A narrative synthesis of all studies will be presented. If feasible, the effectiveness data will be synthesised using appropriate statistical techniques. The methodological quality of the included

For peer review only

studies will be assessed with the Effective Public Health Practice Project quality assessment tool (EPHPP).

Ethics and Dissemination

Ethical approval is not required for this study. Findings will be disseminated widely via peer-reviewed publications and presentations at conferences related to this field.

Registration Details

PROSPERO CRD42022385547

STRENGTHS AND LIMITATIONS

- This PRISMA-guided review applies a systematic methodology that includes a comprehensive search strategy and well-defined eligibility criteria for screening.
- We follow a dual-review process; a second independent researcher is employed throughout the screening and data extraction process.
- This review uses the Effective Public Health Practice Project quality assessment tool (EPHPP) – a tool validated for reviews of intervention effectiveness – to assess the methodological quality of included studies and comment on the strength of the existing evidence.
- The heterogeneity of the interventions and tools used to measure emotion regulation may not allow for direct comparisons between studies as part of a planned meta-analysis.

For peer review only

INTRODUCTION

Approximately 75% of mental health problems begin in adolescence [1]. In the UK, 1 in 6 adolescents (aged 11-16 years) have been identified as having a probable mental health problem – a figure which has steadily increased since 2004 [2]. Current attempts to support this population are designed to target specific conditions (e.g., depression). However, 60% of those adolescents with one diagnosable mental health problem have one or more additional conditions [3].

Mental health comorbidity – the presence of two or more mental illnesses in an individual - is the rule, rather than the exception, and has been associated with greater clinical severity and a greater detriment to overall quality of life [4]. There is also significant overlap in the aetiological origins of different psychopathologies, including evidence of major overlap in both genetic and environmental contributors between most disorders (e.g., major depressive disorder, bipolar disorder, and schizophrenia) [5]. Further complexity emerges from evidence linking non-specific risk factors (such as childhood adverse experiences: ACEs) with the onset of psychopathology [6]. ACEs (e.g., abuse and domestic violence) are a robust predictor of various mental and physical disorders and symptoms [7,8]. Whilst the mechanisms underpinning the aetiology and development of different psychopathologies are complex, associations have been found across a range of different conditions, including mood disorders, anxiety disorders, and substance use disorders [6].

A Transdiagnostic Approach

Given this, researchers have highlighted the need to understand and develop interventions that are directly effective across several disorders, or which alter psychopathological processes common to multiple conditions; these transdiagnostic strategies would offer broader, more effective support for adolescents struggling with their mental health [9]. The Unified Protocol for the transdiagnostic treatment of emotional disorders (UP) is one such strategy, which targets the emotional processes (e.g., emotional reactivity, emotional responding) underlying multiple different disorders. The UP is a transdiagnostic, emotion-focused cognitive-behavioural therapy (CBT) designed to treat all anxiety and

For peer review only

For peer review only - http://bmjopen.bmj.com/site/about/guidelines.xhtml

unipolar mood disorders, as well as other disorders with strong emotional components (e.g., dissociative and somatoform disorders [10]). Preliminary evidence has found it an effective treatment for adolescents with emotional disorders [11, 12], demonstrating the possible utility of transdiagnostic approaches in adolescent mental health.

Emerging evidence suggests the transdiagnostic approach could be effective in targeting a range of psychopathologies in young people and can activate a range of related, beneficial developmental cascades, including improvement in social and academic outcomes in adolescence, as well as improved sleeping patterns [13]. A transdiagnostic approach to treatment is also thought to be time- and cost-effective compared to disorder-specific strategies and may offer a more sustainable alternative to treatments currently available to this population [4].

Emotion Regulation – A Transdiagnostic Target?

Emotion regulation has received increased attention in recent years as a transdiagnostic mechanism and clinical target in psychological treatment [14]. Emotion regulation is a multidimensional process wherein an individual monitors, evaluates and shapes their emotions, when they have them, and how they experience or express them. This process is typically understood to be goal-directed, helping individuals to meet the demands of their environment and achieve their ambitions (e.g. remaining calm to resolve a conflict) [15]. Gross provides a well-defined 'Process model of emotion regulation' [16,17] of specific relevance for research and practice, whereby an individual recognises an emotion regulation goal, selects, and finally implements specific emotion regulation strategies [18]. Gross defined a set of five distinct emotion regulation strategies occurring at different points in an emotional experience: situation selection, situation modification, attentional deployment, cognitive change, and response modulation [18]. Situation selection can be understood as an individual's efforts to alter the likelihood of being in an emotion-evoking situation. Situation modification involves modifying a situation at the time to change its emotional impact. Attentional deployment involves directing attention toward or away from an emotion or its causes. Cognitive change enables reappraising a situation to change its emotional significance. Response modulation

For peer review only - http://bmjopen.bmj.com/site/about/guidelines.xhtml

includes any efforts to modify the behavioural, experiential, and physiological elements of an emotional response. Each of these strategies can be understood to influence an individual's emotional response in a way that can be interpreted as adaptive (e.g., problem-solving, acceptance) or maladaptive (e.g., withdrawal, suppression) [17], depending on the context.

Maladaptive patterns of emotional experience or expression that interfere with goal-directed activity are typically understood as emotion *dysregulation* and have physiological, cognitive, and social consequences [17,18]. Emotion dysregulation can also be understood to represent problematic emotion dynamics: persistence, lability, and intensity of emotions [19]. Evidence demonstrates emotion dysregulation is present across a range of psychopathologies, including internalising (e.g., generalised anxiety disorder, major depressive disorder, dysthymia) and externalising disorders (e.g., attention-deficit/hyperactivity disorder, conduct disorder, oppositional defiant disorder) [20]. For example, generalised anxiety disorder has been associated with a lack of understanding of emotions and an increased reliance on emotion regulation strategies that could be understood as maladaptive, such as withdrawal [20]. Similarly, attention-deficit/hyperactivity disorder (ADHD) is characterised by individuals' emotion regulation deficits and emotion reactivity [21].

Most evidence on the impact of emotion regulation on psychopathology has been derived from adult populations [22]. However, recent findings are beginning to show a close association between emotion regulation and psychopathology in adolescence [23]. Evidence indicates a significant shift in emotion regulation between ages 13 and 15 (e.g., access to strategies, use of adaptive vs. maladaptive strategies), suggesting that adolescence is a particularly vulnerable period in the development of emotion regulation [24]. Therefore, interventions targeting emotion regulation as a transdiagnostic construct central to developing and maintaining psychopathology may reduce the risk and severity of adolescent mental health problems.

Emotion Regulation Interventions

For peer review only - http://bmjopen.bmj.com/site/about/guidelines.xhtml

Existing psychological interventions adopt different approaches to improving emotional regulation. Some focus on reducing the use of maladaptive strategies such as rumination (e.g., Rumination-focused cognitive behavioural therapy; RF-CBT [25]), while others focus on increasing the use of adaptive strategies such as acceptance (e.g., Acceptance and Commitment Therapy [26]). Both the adult and adolescent literature indicate that such interventions improve emotion regulation and mediate decreases in psychopathological symptoms within certain diagnoses (e.g., anxiety) [27, 23]. However, the effectiveness of these interventions in improving multiple mental health and functional outcomes, rather than specific and individual symptom groups, is largely unknown [28].

Further, much of the research to date has focused on the delivery of such interventions in person despite a growing number of digital solutions for adolescent emotion regulation and psychopathology [23]. Previous research examining the effectiveness of several different digital interventions targeting emotion regulation in adolescents found that, in general, such interventions (e.g., digital games and biofeedback) can be effective in improving emotion regulation [29]. Digital interventions also have a greater capacity for innovation and engagement with adolescents [30] and the potential to extend effective care cost-effectively and sustainably [31], but less is known about how such interventions can be applied at scale to support this population.

A Self-Directed, Digital Approach

An increasing number of innovative *self-directed* digital interventions are being developed, which target emotion regulation and related processes (e.g. emotional self-awareness) [32,33]. Such interventions (e.g., mobile apps) are led by the service user, with little to no support from anyone else (e.g., therapist, parent/carer), and aim to widen access to support for adolescents. Despite a growing number of self-directed digital interventions made available to adolescents, the effectiveness of interventions delivered in this self-directed and digital format for improving emotion regulation and psychopathology is, as yet, unclear. Further, more evidence is needed to determine the 'active

For peer review only - http://bmjopen.bmj.com/site/about/guidelines.xhtml

ingredients' of self-directed digital interventions that most improve emotion regulation and reduce psychopathology in adolescents [34]. As the prevalence of adolescent mental health problems increases, innovative digital solutions could help to ensure that a broad range of adolescents can access and engage in support for their mental health.

Review Objectives

To address some of these issues, the proposed systematic review will synthesise evidence on current self-directed digital interventions available to adolescents (aged 11-18 years) to investigate their effectiveness in addressing emotion (dys-)regulation, psychopathology, and functioning (e.g., academic achievement).

Despite an increase in the number of available self-directed digital interventions for emotion regulation, evidence about their effectiveness among adolescent populations has yet to be synthesised. This review provides an important extension to existing work which has thus far demonstrated the effectiveness of in-person or therapist-supported emotion regulation interventions available for young people (aged 6 – 24 years) [20], as well as the utility of a broad spectrum of digital emotion regulation interventions for adolescents [29]. This review takes a more specific focus to develop evidence on a burgeoning number of *self-directed*, scalable, digital mental health interventions available to adolescents with or without diagnosed psychopathology. Specifically, we will seek to answer the following research questions:

1. Are current self-directed digital interventions that target emotion regulation effective in improving emotion (dys-)regulation in adolescents?
2. Are these interventions effective in treating psychopathology and improving function (e.g. academic achievement)?
3. What are the specific components (e.g. mood monitoring) of these interventions that most improve emotion (dys-)regulation and/or psychopathology and functioning in adolescents?

For peer review only - http://bmjopen.bmj.com/site/about/guidelines.xhtml

METHODS AND ANALYSIS

Protocol and Registration

This protocol is written in accordance with the Preferred Reporting Items for Systematic Review and Meta-Analysis Protocols (PRISMA-P) guidelines [35]. The completed PRISMA-P checklist is provided in *Supplementary File 1*. The systematic review has been registered in PROSPERO, the International Prospective Register of Systematic Reviews (CRD42022385547). Important amendments to this protocol will be reported and published with the review results.

Eligibility Criteria

The following criteria (*Table 1*) are set out to screen identified studies using a PICOS framework [36].

Table 1. *Inclusion and Exclusion Criteria*

	Inclusion	Exclusion
1. Population	• Any adolescent (aged 11-18)<br>• With or without a diagnosed mental health problem<br>• Neurotypical/neurodiverse	• Adolescents outside the specified age range<br>• Non-human participants
2. Intervention	• Any mental health intervention delivered entirely by digital	• The intervention is not entirely self-directed (i.e. it includes modules, or components which are

For peer review only

	technology (e.g. app-based, web-based etc.)<br>• Can be delivered in any appropriate setting such as at home, in school, in voluntary and community settings, primary, secondary, or tertiary care<br>• Must address emotion (dys-) regulation, but this need not be the focus<br>• Must be self-directed (i.e. the intervention is completed entirely by the adolescent, with no support from a clinician, teacher, parent, or caregiver)	not completed by the adolescent, or are completed with support from a clinician, teacher, parent, or caregiver)
3. Comparison	• Employing a control group is not an essential criterion for inclusion in the review.	• Employing a control group is not an essential criterion for inclusion in the review.
4. Outcomes	• Must measure emotion (dys-) regulation using a valid or appropriate measure	• If a valid and appropriate item, scale, or measure is not used, the authors of the paper must comment on their reasoning for choosing their measure
5. Study	• Publication dates from 2010 to present	• Non-English language

For peer review only - http://bmjopen.bmj.com/site/about/guidelines.xhtml

	• Studies from any geographical location<br>• Primary research study with direct contact or observation of individuals<br>• Grey literature, theses, reports, pre-prints	• Descriptive studies such as case reports/studies that do not include direct contact or observation of individuals<br>• Review papers, books, editorials, commentaries, poster abstracts, conferences

For peer review only

Information Sources

Ten electronic databases will be searched for studies published any time from January 2010: MEDLINE, PsycINFO; Global Health: Scopus; Web of Science Core Collection; EBSCO CINAHL; EBSCO ERIC; OVID Embase; The Cochrane Central Register of Controlled Trials (CENTRAL); and ACM Digital Library. Grey literature such as preprints and theses are also included in this review (databases: HMIC, EThOS, Psyarxiv, Trip, ClinicalTrials.gov). This search will be updated periodically to identify any new relevant research from the selected databases before the results are finalised (most recent search August 2023).

Search Strategy

The search strategy (*Supplementary File 2*) is designed to identify all studies examining one or more self-directed digital mental health interventions for adolescents, which include an emotion (dys-) regulation component. It will search for synonyms for the following three concepts: *adolescents, emotion (dys-) regulation, and self-directed digital interventions*. The search will be limited to English language studies conducted with a human population.

Study Selection

All identified studies will be exported to Endnote 20, and any duplicates will be removed following a specified method [37] before being imported into Rayyan [38] for further deduplication and

screening. Abstracts and titles will be screened based on the inclusion and exclusion criteria (**Table 1**). Those studies that meet these criteria will enter the full-text screening stage for further checks against the eligibility criteria. The full-text screening will allow for the identification of those interventions that do not address emotion (dys-)regulation directly. A second researcher (E.L.) will be assigned a random 10% of the identified studies for screening at the title and abstract stage. Discrepancies throughout the study selection process will be resolved through consensus between AT and EL. If discrepancies cannot be resolved through discussion, a third reviewer will be consulted to adjudicate.

Data Extraction and Management

Data will be extracted and collated by two independent reviewers (A.T. and E.L.) and tracked in Microsoft Excel using a structured coding form and associated coding manual. The form will be piloted on a sample of included studies and refined before extraction begins. Information relating to study characteristics (e.g. author(s), publication date), participant characteristics (e.g. age, gender), digital intervention characteristics (e.g. name, focus), and relevant clinical and emotion dysregulation outcomes will be extracted from each study. Information to determine any study bias will also be collated. Study investigators will be contacted for missing/unreported data or additional details. If we are not able to obtain sufficient details/raw data using this approach, we will exclude the respective studies.

Outcomes

The primary outcome of this review is the change in emotion (dys-)regulation, psychopathology, and functioning (e.g. academic achievement) occurring as a result of participation in a self-directed digital intervention that addresses emotion (dys-) regulation. Emotion (dys-)regulation must be assessed, and where possible, using a valid and appropriate item, scale, or measure (e.g., Child Social Behaviour Questionnaire) [39], including through clinical interviews or self-reported measures. An existing review of emotion (dys-)regulation assessment [40] and similar reviews [22,41] will be used as guidance to decide on a measure's eligibility. Symptoms of psychopathology will be assessed

For peer review only - http://bmjopen.bmj.com/site/about/guidelines.xhtml

For peer review only

by any available valid and appropriate measure, including through clinical interview or self-reported measures.

Quality and Risk of Bias Assessment

Two researchers (A.T. and E.L.) will independently assess the methodological quality of the included studies using the Effective Public Health Practice Project Quality Assessment tool (EPHPP). The EPHPP is applicable to a range of quantitative study designs (e.g. case-control studies) and has been judged to be particularly suitable for systematic reviews on the effectiveness of interventions/treatments [42]. Evidence has shown the EPHPP has good content and construct validity [42,43]. The AACODS Checklist will be used to assess the quality and content of any included grey literature (i.e., theses and reports). It has been designed as a critical appraisal tool specifically for use with grey literature sources.

If sufficient data is available, potential reporting bias will be assessed visually using a funnel plot and commented on in the review. An asymmetrical plot suggests a publication bias [44]. If asymmetry in the funnel plot is detected, the studies will be reviewed to assess whether this asymmetry was likely due to publication bias or other factors such as methodological or clinical heterogeneity.

Data Synthesis

Information from the included studies will be synthesised in line with guidance from Popay [45]. A narrative overview of the study characteristics and methodology, participant characteristics and the digital interventions used to address emotion (dys-)regulation will be included. A summary of any observed changes in emotion dysregulation and/or psychopathology occurring as a result of the intervention will be provided. Additionally, any general improvements in functioning (e.g., social or academic) that occur as a result of participation in the intervention will also be described if reported in the included papers.

For peer review only - http://bmjopen.bmj.com/site/about/guidelines.xhtml

Given the findings of preliminary searches and previous reviews [23], it is anticipated that there will be high heterogeneity across studies. However, a meta-analysis will be conducted if (a) there are a minimum of two papers included in the review and (b) the available data within the included studies is sufficiently homogenous (Q statistic is non-significant and/or $I^2 < 25\%$). Studies judged to be of low quality will be excluded from the meta-analysis. Two meta-analyses are planned: one with psychopathology as the primary outcome and one with emotion dysregulation as the primary outcome. Outcomes will be pooled using a random-effects model, in line with current recommendations for meta-analysis models in psychology [46].

Subgroup analyses will be completed if sufficient data is available to explore the efficacy of different intervention components (i.e. mood monitoring, psychoeducation) in improving emotion dysregulation and psychopathology in adolescents. Subgroup analyses will also be conducted to identify whether there are differences in effect size or heterogeneity due to study-level factors (i.e. quality). These analyses will be conducted when $I^2 < 50\%$ and there are ten or more studies ($n > 10$) to evaluate differences by specific a priori subgroups.

All analyses will be conducted in RStudio v2023.06.0+421 for macOS Sonoma 14.1.2 (meta, metasen, metafor packages)..

Patient and Public Involvement

Since this is a systematic review protocol, no patients or the public were involved.

Ethics and Dissemination

Due to the characteristics of this study design, ethical approval was not required for this study. The findings of this systematic review will be disseminated widely via peer-reviewed publications and presentations at conferences related to this field.

For peer review only - http://bmjopen.bmj.com/site/about/guidelines.xhtml

Amendments

Any amendments to this protocol will be documented and reported in the final peer-reviewed publication.

AUTHORS' CONTRIBUTIONS

AT is the guarantor. AT, GH and BO conceived the initial idea for the systematic review and design of the protocol. AT drafted the manuscript, and GH and BO provided critical insight. AT, GH, BO, EL and BW contributed to the revision of the manuscript and approved the final version.

For peer review only

FUNDING STATEMENT

This work is funded by the UK Research and Innovation, Economic and Social Research Council (grant number: ES/P000703/1).

COMPETING INTERESTS STATEMENT

None declared.

REFERENCES

1. Kim-Cohen J, Caspi A, Moffitt TE, *et al*. Prior juvenile diagnoses in adults with mental disorder: developmental follow-back of a prospective-longitudinal cohort. Archives of general psychiatry. 2003 Jul 1;60(7):709-17. doi.org/10.1001/archpsyc.60.7.709
2. Newlove-Delgado, T., Williams, T., Robertson, K., *et al*. Mental Health of Children and Young People in England 2021-wave 2 follow up to the 2017 survey. 2021. Retrieved November 25, 2021, from https://digital.nhs.uk/dataandinformation/publications/statistical/mental-health-of-childrenand-young-people-in-england/2020-wave-1-follow-up.
3. World Health Organization. Adolescent mental health. 2021. Retrieved November 25, 2021, from https://www.who.int/news-room/factsheets/detail/adolescent-mental-health

For peer review only - http://bmjopen.bmj.com/site/about/guidelines.xhtml

4. Dalgleish T, Black M, Johnston D, *et al*. Transdiagnostic approaches to mental health problems: Current status and future directions. Journal of consulting and clinical psychology. 2020 Mar;88(3):179. doi.org/10.1037/ccp0000482

5. Uher R, Zwicker A. Etiology in psychiatry: embracing the reality of poly-gene-environmental causation of mental illness. World psychiatry. 2017 Jun;16(2):121-9. doi.org/10.1002/wps.20436

6. Keyes KM, Eaton NR, Krueger RF, *et al*. Childhood maltreatment and the structure of common psychiatric disorders. The British Journal of Psychiatry. 2012 Feb;200(2):107-15. doi.org/10.1192/bjp.bp.111.093062

7. Hosang GM, Johnson SL, Kiecolt-Glaser J, *et al*. Gender specific association of child abuse and adult cardiovascular disease in a sample of patients with Basal Cell Carcinoma. Child Abuse & Neglect. 2013 Jun 1;37(6):374-9. doi.org/10.1016/j.chiabu.2012.09.018

8. Fritz J, De Graaff AM, Caisley H, *et al*. A systematic review of amenable resilience factors that moderate and/or mediate the relationship between childhood adversity and mental health in young people. Frontiers in psychiatry. 2018 Jun 19;9:230. doi.org/10.3389/fpsyt.2018.00230

9. Roefs A, Fried EI, Kindt M, *et al*. A new science of mental disorders: Using personalised, transdiagnostic, dynamical systems to understand, model, diagnose and treat psychopathology. Behaviour Research and Therapy. 2022 Jun 1;153:104096. doi.org/10.1016/j.brat.2022.104096

10. Farchione TJ, Fairholme CP, Ellard KK, *et al*. Unified protocol for transdiagnostic treatment of emotional disorders: a randomized controlled trial. Behavior therapy. 2012 Sep 1;43(3):666-78. doi.org/10.1016/j.beth.2012.01.001

11. Ehrenreich-May J, Rosenfield D, Queen AH, *et al*. An initial waitlist-controlled trial of the unified protocol for the treatment of emotional disorders in adolescents. Journal of Anxiety Disorders. 2017 Mar 1;46:46-55. doi.org/10.1016/j.janxdis.2016.10.006

12. Carlucci L, Saggino A, Balsamo M. On the efficacy of the unified protocol for transdiagnostic treatment of emotional disorders: A systematic review and meta-analysis. Clinical Psychology Review. 2021 Jul 1;87:101999. doi.org/10.1016/j.cpr.2021.101999

For peer review only

13. Forbes MK, Rapee RM, Krueger RF. Opportunities for the prevention of mental disorders by reducing general psychopathology in early childhood. Behaviour research and therapy. 2019 Aug 1;119:103411. doi.org/10.1016/j.brat.2019.103411

14. Aldao A, Gee DG, De Los Reyes A, *et al*. Emotion regulation as a transdiagnostic factor in the development of internalizing and externalizing psychopathology: Current and future directions. Development and psychopathology. 2016 Nov;28(4pt1):927-46. doi.org/10.1017/S0954579416000638

15. D'Agostino A, Covanti S, Rossi Monti M, *et al*. Reconsidering emotion dysregulation. Psychiatric Quarterly. 2017 Dec;88:807-25. doi.org/10.1007/s11126-017-9499-6

16. Eftekhari A, Zoellner LA, Vigil SA. Patterns of emotion regulation and psychopathology. Anxiety, Stress, & Coping. 2009 Oct 1;22(5):571-86. doi.org/10.1080/10615800802179860

17. Gross JJ. Antecedent-and response-focused emotion regulation: divergent consequences for experience, expression, and physiology. Journal of personality and social psychology. 1998 Jan;74(1):224. doi.org/10.1037/0022-3514.74.1.224

18. Gross JJ. Emotion regulation in adulthood: Timing is everything. Current directions in psychological science. 2001 Dec;10(6):214-9. doi.org/10.1111/1467-8721.00152

19. Thompson RA. Emotion dysregulation: A theme in search of definition. Development and psychopathology. 2019 Aug;31(3):805-15. doi.org/ 10.1017/S0954579419000282

20. Helland SS, Mellblom AV, Kjøbli J, *et al*. Elements in mental health interventions associated with effects on emotion regulation in adolescents: a meta-analysis. Administration and Policy in Mental Health and Mental Health Services Research. 2022 Nov;49(6):1004-18. doi.org/10.1007/s10488-022-01213-2

21. Christiansen H, Hirsch O, Albrecht B, Chavanon ML. Attention-deficit/hyperactivity disorder (ADHD) and emotion regulation over the life span. Current psychiatry reports. 2019 Mar;21:1-1. doi.org/10.1007/s11920-019-1003-6

22. Aldao A, Nolen-Hoeksema S, Schweizer S. Emotion-regulation strategies across psychopathology: A meta-analytic review. Clinical psychology review. 2010 Mar 1;30(2):217-37. doi.org/10.1016/j.cpr.2009.11.004

For peer review only

23. Moltrecht B, Deighton J, Patalay P, *et al*. Effectiveness of current psychological interventions to improve emotion regulation in youth: a meta-analysis. European child & adolescent psychiatry. 2021 Jun;30(6):829-48. doi.org/10.1007/s00787-020-01498-4

24. Zimmermann P, Iwanski A. Emotion regulation from early adolescence to emerging adulthood and middle adulthood: Age differences, gender differences, and emotion-specific developmental variations. International journal of behavioral development. 2014 Mar;38(2):182-94. doi.org/10.1177/0165025413515405

25. Watkins E, Scott J, Wingrove J, *et al*. Rumination-focused cognitive behaviour therapy for residual depression: A case series. Behaviour research and therapy. 2007 Sep 1;45(9):2144-54. doi.org/10.1016/j.brat.2006.09.018

26. Hayes SC, Strosahl KD, Wilson KG. Acceptance and commitment therapy: American Psychological Association. Washington, DC.[Google Scholar]. 2009.

27. Gratz KL, Weiss NH, Tull MT. Examining emotion regulation as an outcome, mechanism, or target of psychological treatments. Current opinion in psychology. 2015 Jun 1;3:85-90. doi.org/10.1016/j.copsyc.2015.02.010

28. Andersen P, Toner P, Bland M, *et al*. Effectiveness of transdiagnostic cognitive behaviour therapy for anxiety and depression in adults: a systematic review and meta-analysis. Behavioural and cognitive psychotherapy. 2016 Nov;44(6):673-90. doi.org/10.1017/S1352465816000229

29. Reynard, S., Dias, J., Mitic, M., Schrank, B. and Woodcock, K.A., 2022. Digital interventions for emotion regulation in children and early adolescents: systematic review and meta-analysis. JMIR Serious Games, 10(3), p.e31456.

30. Liverpool S, Mota CP, Sales CM, *et al*. Engaging children and young people in digital mental health interventions: systematic review of modes of delivery, facilitators, and barriers. Journal of medical Internet research. 2020 Jun 23;22(6):e16317. doi.org/10.2196/16317

31. Mohr DC, Lyon AR, Lattie EG, *et al*. Accelerating digital mental health research from early design and creation to successful implementation and sustainment. Journal of medical Internet research. 2017 May 10;19(5):e7725. doi.org/10.2196/jmir.7725

For peer review only

32. Moltrecht B, Patalay P, Bear HA, *et al*. Interdisciplinary development of a transdiagnostic mobile app to enhance children's emotion regulation: sharing insights and lessons learned. JMIR Formative Research. 2021 Nov 16. doi.org/10.2196/28300

33. Moltrecht B, Patalay P, Deighton J, *et al*. A school-based mobile app intervention for enhancing emotion regulation in children: exploratory trial. JMIR mHealth and uHealth. 2021 Jul 14;9(7):e21837. doi.org/10.2196/21837

34. Braet C, Theuwis L, Van Durme K, *et al*. Emotion regulation in children with emotional problems. Cognitive Therapy and Research. 2014 Oct;38:493-504. doi.org/10.1007/s10608-014-9616-x

35. Moher D, Shamseer L, Clarke M, *et al*, PRISMA-P Group. Preferred reporting items for systematic review and meta-analysis protocols (PRISMA-P) 2015 statement. Syst Rev 2015;4(1):1. doi.org/10.1186/2046-4053-4-1

36. Munn Z, Stern C, Aromataris E, *et al*. What kind of systematic review should I conduct? A proposed typology and guidance for systematic reviewers in the medical and health sciences. BMC medical research methodology. 2018 Dec;18(1):1-9. doi.org/10.1186/s12874-017-0468-4

37. Falconer J. Removing duplicates from an endnote library. [Internet]. . 2018 [cited 2022Apr25]. Available from: https://blogs.lshtm.ac.uk/library/2018/12/07/removing-duplicates-from-an-endnote-library/

38. Ouzzani M, Hammady H, Fedorowicz Z, Elmagarmid A. Rayyan—a web and mobile app for systematic reviews. Systematic reviews. 2016 Dec;5:1-0. doi.org/10.1186/s13643-016-0384-4

39. Luteijn EE, Jackson SA, Volkmar FR, *et al*. The development of the Children's Social Behavior Questionnaire: Preliminary data. Journal of Autism and developmental Disorders. 1998 Dec. doi.org/10.1023/A:1026060330122

40. Adrian M, Zeman J, Erdley C, *et al*. Emotional dysregulation, and interpersonal difficulties as risk factors for nonsuicidal self-injury in adolescent girls. Journal of abnormal child psychology. 2011 Apr;39:389-400. doi.org/10.1007/s10802-010-9465-3

41. Sloan E, Hall K, Moulding R, *et al*. Emotion regulation as a transdiagnostic treatment construct across anxiety, depression, substance, eating and borderline personality disorders: A

For peer review only

systematic review. Clinical psychology review. 2017 Nov 1;57:141-63. doi.org/10.1016/j.cpr.2017.09.002

42. Deeks JJ, Dinnes J, D'Amico R, *et al*. Evaluating non-randomised intervention studies. Health technology assessment (Winchester, England). 2003;7(27):iii-173. doi.org/10.3310/hta7270

43. Thomas BH, Ciliska D, Dobbins M, Micucci S. A process for systematically reviewing the literature: providing the research evidence for public health nursing interventions. Worldviews on Evidence-Based Nursing. 2004 Sep;1(3):176-84. doi.org/10.1111/j.1524-475X.2004.04006.x

44. Mavridis D, Salanti G. How to assess publication bias: funnel plot, trim-and-fill method and selection models. BMJ Ment Health. 2014 Feb 1;17(1):30-. doi.org/10.1136/eb-2013-101699

45. Popay J, Roberts H, Sowden A, *et al*. Guidance on the conduct of narrative synthesis in systematic reviews. A product from the ESRC methods programme Version. 2006 Apr 1;1(1):b92.

46. Schmidt FL, Oh IS, Hayes TL. Fixed-versus random-effects models in meta-analysis: Model properties and an empirical comparison of differences in results. British Journal of Mathematical and Statistical Psychology. 2009 Feb;62(1):97-128. doi.org/10.1348/000711007X255327

For peer review only

For peer review only - http://bmjopen.bmj.com/site/about/guidelines.xhtml

For peer review only

For peer review only - http://bmjopen.bmj.com/site/about/guidelines.xhtml

Supplementary File 1

Database: Ovid PsycINFO 1806 to present

Search Strategy:

----

1 (adolescen* or juvenil* or youth* or teen* or pubescen* or pediatric* or paediatric* or "high school" or "secondary school" or school* or "young people" or "young person*" or "young adult*" or "young man" or "young men" or "young woman" or "young women" or student*).ti,ab

2 (emotion* AND (regulat* or dysregulat* or generat* OR modulat* OR adapt* OR cop* OR react* OR arous* OR avoid* OR behavio* OR suppress* OR ruminat*)).,ti,ab

3 (affect AND (regulat* or dysregulat* or generat* OR modulat* OR adapt* OR cop* OR react* OR arous* OR avoid* OR behavio* OR suppress* OR ruminat*)).ti,ab

4 ("self-concept" OR "mood regulat*" OR "situation selection" OR "situation modification" OR "attentional deployment" OR "response modulation" OR "problem focused coping" OR "impulsivity" OR "hyper-reactivity" OR "anger regulation" OR "experiential avoidance" OR "expressive suppression" OR "thought suppression" OR "ruminat*" OR "emotion regulation questionnaire" OR "Difficulties in Emotion Regulation Scale" OR "emotion regulation strategy").ti,ab

5 2 or 3 or 4

6 (treat* or interven* or "early intervention" or "self-directed intervention" or "self-directed" or "public health").ti,ab.

7 phon* or mobile* or "smart-phone*" or device* or computer* or tablet* or "device-based" or "app-based" or apps or app or "sensor*" or "social media").ti,ab.

8 ((digital or virtual or online or interactive) and (health* or intervention or support or platform*)).ti,ab.

9 ("eHealth" or "e-health" or "m-health" or mHealth or "tele-health" or telehealth).ti,ab.

10 7 or 8 or 9

11 1 and 5 and 6 and 10

12 limit 11 to (peer reviewed journal and human and english language and (childhood <birth to 12 years> or adolescence <13 to 17 years>) and ("0100 journal" or "0110 peer-reviewed journal") and journal article and english and human and yr="1995 -Current")

For peer review only

Database: PubMed MEDLINE

Search Strategy:

----

1 adolescen*[Title/Abstract] OR juvenil*[Title/Abstract] OR youth*[Title/Abstract] OR teen*[Title/Abstract] OR pubescen*[Title/Abstract] OR pediatric*[Title/Abstract] OR paediatric*[Title/Abstract] OR "high school"[Title/Abstract] OR "secondary school"[Title/Abstract] OR school*[Title/Abstract] OR "young people"[Title/Abstract] OR "young person*"[Title/Abstract] OR "young adult*"[Title/Abstract] OR "young man"[Title/Abstract] OR "young men"[Title/Abstract] OR "young woman"[Title/Abstract] OR "young women"[Title/Abstract] OR student*[Title/Abstract]

2 emotion*[Title/Abstract] AND (regulat*[Title/Abstract] OR dysregulat*[Title/Abstract] OR generat*[Title/Abstract] OR modulat*[Title/Abstract] OR adapt*[Title/Abstract] OR react*[Title/Abstract] OR arous*[Title/Abstract] OR avoid*[Title/Abstract] OR behavio*[Title/Abstract] OR suppress*[Title/Abstract] OR ruminat*[Title/Abstract])

3 affect[Title/Abstract] AND (regulat*[Title/Abstract] OR dysregulat*[Title/Abstract] OR generat*[Title/Abstract] OR modulat*[Title/Abstract] OR adapt*[Title/Abstract] OR react*[Title/Abstract] OR arous*[Title/Abstract] OR avoid*[Title/Abstract] OR behavio*[Title/Abstract] OR suppress*[Title/Abstract] OR ruminat*[Title/Abstract])

4 "self-concept"[Title/Abstract] OR "mood regulat*"[Title/Abstract] OR "situation selection"[Title/Abstract] OR "situation modification"[Title/Abstract] OR "attentional deployment"[Title/Abstract] OR "response modulation"[Title/Abstract] OR "problem focused coping"[Title/Abstract] OR "impulsivity"[Title/Abstract] OR "hyper-reactivity"[Title/Abstract] OR "anger regulation"[Title/Abstract] OR "experiential avoidance"[Title/Abstract] OR "expressive suppression"[Title/Abstract] OR "thought suppression"[Title/Abstract] OR "ruminat*"[Title/Abstract] OR "emotion regulation questionnaire"[Title/Abstract] OR "Difficulties in Emotion Regulation Scale"[Title/Abstract] OR "emotion regulation strategy"[Title/Abstract]

5 #2 OR #3 OR #4

6 treat*[Title/Abstract] OR interven*[Title/Abstract] OR "early intervention"[Title/Abstract] OR "self-directed intervention"[Title/Abstract] OR "self-directed"[Title/Abstract] OR "public health"[Title/Abstract]

7 phon*[Title/Abstract] OR mobile*[Title/Abstract] OR "smart-phone*"[Title/Abstract] OR device*[Title/Abstract] OR computer*[Title/Abstract] OR tablet*[Title/Abstract] OR "device-based"[Title/Abstract] OR "app-based"[Title/Abstract] OR apps[Title/Abstract] OR app[Title/Abstract] OR "sensor*"[Title/Abstract] OR "social media"[Title/Abstract]

8 ((digital[Title/Abstract] OR virtual[Title/Abstract] OR online[Title/Abstract] OR interactive[Title/Abstract]) AND (health*[Title/Abstract] OR intervention[Title/Abstract] OR support[Title/Abstract] OR platform*[Title/Abstract]))

For peer review only

9 "eHealth"[Title/Abstract] OR "e-health"[Title/Abstract] OR "m-health"[Title/Abstract] OR mHealth[Title/Abstract] OR "tele-health"[Title/Abstract] OR telehealth[Title/Abstract]

10 #7 OR #8 OR #9

11 #1 and #5 and #6 and #10

12 #1 and #5 and #6 and #10 Filters: Humans, English, MEDLINE, from 1995 – 2023

For peer review only

Database: Scopus

Search Strategy:

(TITLE-ABS-KEY (adolescen* OR juvenil* OR youth* OR teen* OR pubescen* OR pediatric* OR paediatric* OR "high school" OR "secondary school" OR school* OR "young people" OR "young person*" OR "young adult*" OR "young man" OR "young men" OR "young woman" OR "young women" OR student*) AND TITLE-ABS-KEY ((emotion* AND (regulat* OR dysregulat* OR generat* OR modulat* OR adapt* OR cop* OR react* OR arous* OR avoid* OR behavio* OR suppress* OR ruminat*)) OR (affect AND (regulat* OR dysregulat* OR generat* OR modulat* OR adapt* OR cop* OR react* OR arous* OR avoid* OR behavio* OR suppress* OR ruminat*)) OR (self-concept OR "mood regulat*" OR "situation selection" OR "situation modification" OR "attentional deployment" OR "response modulation" OR "problem focused coping" OR impulsivity OR hyper-reactivity OR "anger regulation" OR "experiential avoidance" OR "expressive suppression" OR "thought suppression" OR ruminat* OR "emotion regulation questionnaire" OR "Difficulties in Emotion Regulation Scale" OR "emotion regulation strategy")) AND TITLE-ABS-KEY (treat* OR interven* OR "early intervention" OR "self-directed intervention" OR "self-directed" OR "public health") AND TITLE-ABS-KEY ((phon* OR mobile* OR "smart-phone*" OR device* OR computer* OR tablet* OR "device-based" OR "app-based" OR apps OR app OR "sensor*" OR "social media") OR ((digital OR virtual OR online OR interactive) AND (health* OR intervention OR support OR platform*)) OR ("eHealth" OR "e-health" OR "m-health" OR mhealth OR "tele-health" OR telehealth))) AND ORIG-LOAD-DATE AFT 20230203 AND (LIMIT-TO (SUBJAREA , "MEDI") OR LIMIT-TO (SUBJAREA , "SOCI") OR LIMIT-TO (SUBJAREA , "MULT") OR LIMIT-TO (SUBJAREA , "PSYC") OR LIMIT-TO (SUBJAREA , "NURS") OR LIMIT-TO (SUBJAREA , "HEAL")) AND (LIMIT-TO (DOCTYPE , "ar")) AND (LIMIT-TO (EXACTKEYWORD , "Human") OR LIMIT-TO (EXACTKEYWORD , "Humans") OR LIMIT-TO (EXACTKEYWORD , "Adolescent")) AND (LIMIT-TO (LANGUAGE , "English")) AND (EXCLUDE (SUBJAREA , "MEDI") OR EXCLUDE (SUBJAREA , "NEUR") OR EXCLUDE (SUBJAREA , "BIOC") OR EXCLUDE (SUBJAREA , "ARTS") OR EXCLUDE (SUBJAREA , "ENVI") OR EXCLUDE (SUBJAREA , "PHAR")) AND (EXCLUDE (SUBJAREA , "AGRI") OR EXCLUDE (SUBJAREA , "BUSI") OR EXCLUDE (SUBJAREA , "COMP") OR EXCLUDE (SUBJAREA , "DENT") OR EXCLUDE (SUBJAREA , "ENGI")) AND (EXCLUDE (SUBJAREA , "VETE"))

For peer review only

Database: Global Health 1973 to 2022 Week 50

Search Strategy:

----

1 (adolescen* or juvenil* or youth* or teen* or pubescen* or pediatric* or paediatric* or "high school" or "secondary school" or school* or "young people" or "young person*" or "young adult*" or "young man" or "young men" or "young woman" or "young women" or student*).ti,ab

2 (emotion* AND (regulat* or dysregulat* or generat* OR modulat* OR adapt* OR cop* OR react* OR arous* OR avoid* OR behavio* OR suppress* OR ruminat*)).,ti,ab

3 (affect AND (regulat* or dysregulat* or generat* OR modulat* OR adapt* OR cop* OR react* OR arous* OR avoid* OR behavio* OR suppress* OR ruminat*)).,ti,ab

4 ("self-concept" OR "mood regulat*" OR "situation selection" OR "situation modification" OR "attentional deployment" OR "response modulation" OR "problem focused coping" OR "impulsivity" OR "hyper-reactivity" OR "anger regulation" OR "experiential avoidance" OR "expressive suppression" OR "thought suppression" OR "ruminat*" OR "emotion regulation questionnaire" OR "Difficulties in Emotion Regulation Scale" OR "emotion regulation strategy").ti,ab

5 2 or 3 or 4

6 (treat* or interven* or "early intervention" or "self-directed intervention" or "self-directed" or "public health").ti,ab.

7 phon* or mobile* or "smart-phone*" or device* or computer* or tablet* or "device-based" or "app-based" or apps or app or "sensor*" or "social media").ti,ab.

8 ((digital or virtual or online or interactive) and (health* or intervention or support or platform*)).ti,ab.

9 ("eHealth" or "e-health" or "m-health" or mHealth or "tele-health" or telehealth).ti,ab.

10 7 or 8 or 9

11 1 and 5 and 6 and 10

12 limit 11 to (english language and english and (journal or journal article) and yr="1995 -Current")

For peer review only

Database: EBSCO CINAHL

Search Strategy:

----

1 AB adolescen* or juvenil* or youth* or teen* or pubescen* or pediatric* or paediatric* or "high school" or "secondary school" or school* or "young people" or "young person*" or "young adult*" or "young man" or "young men" or "young woman" or "young women" or student*

2 AB emotion* AND (regulat* or dysregulat* or generat* OR modulat* OR adapt* OR cop* OR react* OR arous* OR avoid* OR behavio* OR suppress* OR ruminat*)

3 AB affect AND (regulat* or dysregulat* or generat* OR modulat* OR adapt* OR cop* OR react* OR arous* OR avoid* OR behavio* OR suppress* OR ruminat*)

4 AB "self-concept" OR "mood regulat*" OR "situation selection" OR "situation modification" OR "attentional deployment" OR "response modulation" OR "problem focused coping" OR "impulsivity" OR "hyper-reactivity" OR "anger regulation" OR "experiential avoidance" OR "expressive suppression" OR "thought suppression" OR "ruminat*" OR "emotion regulation questionnaire" OR "Difficulties in Emotion Regulation Scale" OR "emotion regulation strategy"

5 S2 or S3 or S4

6 AB treat* or interven* or "early intervention" or "self-directed intervention" or "self-directed" or "public health"

7 AB phon* or mobile* or "smart-phone*" or device* or computer* or tablet* or "device-based" or "app-based" or apps or app or "sensor*" or "social media"

8 AB (digital or virtual or online or interactive) and (health* or intervention or support or platform*))

9 AB "eHealth" or "e-health" or "m-health" or mHealth or "tele-health" or telehealth

10 S7 or S8 or S9

11 S1 and S5 and S6 and S10

Limiters - Published Date: 19950101-; English Language; Peer Reviewed; Research Article; Human; Publication Type: Journal Article; Age Groups: Child: 6-12 years, Adolescent: 13-18 years; Language: English

For peer review only

Database: EBSCO ERIC

Search Strategy:

----

1 AB adolescen* or juvenil* or youth* or teen* or pubescen* or pediatric* or paediatric* or "high school" or "secondary school" or school* or "young people" or "young person*" or "young adult*" or "young man" or "young men" or "young woman" or "young women" or student*

2 AB emotion* AND (regulat* or dysregulat* or generat* OR modulat* OR adapt* OR cop* OR react* OR arous* OR avoid* OR behavio* OR suppress* OR ruminat*)

3 AB affect AND (regulat* or dysregulat* or generat* OR modulat* OR adapt* OR cop* OR react* OR arous* OR avoid* OR behavio* OR suppress* OR ruminat*)

4 AB "self-concept" OR "mood regulat*" OR "situation selection" OR "situation modification" OR "attentional deployment" OR "response modulation" OR "problem focused coping" OR "impulsivity" OR "hyper-reactivity" OR "anger regulation" OR "experiential avoidance" OR "expressive suppression" OR "thought suppression" OR "ruminat*" OR “emotion regulation questionnaire” OR “Difficulties in Emotion Regulation Scale” OR “emotion regulation strategy”

5 S2 or S3 or S4

6 AB treat* or interven* or "early intervention" or "self-directed intervention" or "self-directed" or "public health"

7 AB phon* or mobile* or "smart-phone*" or device* or computer* or tablet* or "device-based" or "app-based" or apps or app or "sensor*" or "social media"

8 AB (digital or virtual or online or interactive) and (health* or intervention or support or platform*)

9 AB "eHealth" or "e-health" or "m-health" or mHealth or "tele-health" or telehealth

10 S7 or S8 or S9

11 S1 and S5 and S6 and S10

Limiters - Published Date: 19950101-; English Language; Peer Reviewed; Research Article; Human; Publication Type: Journal Article; Age Groups: Child: 6-12 years, Adolescent: 13-18 years; Language: English

For peer review only

Database: The Cochrane Central Register of Controlled Trials (CENTRAL)

Search Strategy:

----

1 adolescen* or juvenil* or youth* or teen* or pubescen* or pediatric* or paediatric* or "high school" or "secondary school" or school* or "young people" or "young person*" or "young adult*" or "young man" or "young men" or "young woman" or "young women" or student*

2 emotion* AND (regulat* or dysregulat* or generat* OR modulat* OR adapt* OR cop* OR react* OR arous* OR avoid* OR behavio* OR suppress* OR ruminat*)

3 affect AND (regulat* or dysregulat* or generat* OR modulat* OR adapt* OR cop* OR react* OR arous* OR avoid* OR behavio* OR suppress* OR ruminat*)

4 self-concept OR "mood regulat*" OR "situation selection" OR "situation modification" OR "attentional deployment" OR "response modulation" OR "problem focused coping" OR "impulsivity" OR hyper-reactivity OR "anger regulation" OR "experiential avoidance" OR "expressive suppression" OR "thought suppression" OR ruminat* OR “emotion regulation questionnaire” OR “Difficulties in Emotion Regulation Scale” OR “emotion regulation strategy”

5 #2 or #3 or #4

6 treat* or interven* or "early intervention" or "self-directed intervention" or "self-directed" or "public health"

7 phon* or mobile* or "smart-phone*" or device* or computer* or tablet* or "device-based" or "app-based" or apps or app or "sensor*" or "social media"

8 (digital or virtual or online or interactive) and (health* or intervention or support or platform*)

9 "eHealth" or "e-health" or "m-health" or mHealth or "tele-health" or telehealth

10 #7 or #8 or #9

11 #1 and #5 and #6 and #10

with Publication Year from 1995 to 2023, in Trials with Public Health, Developmental, Psychosocial and Learning Problems, Child Health in Cochrane Groups

For peer review only

Database: Web of Science: Core Collection

Search Strategy:

----

1 AB=(adolescen* or juvenil* or youth* or teen* or pubescen* or pediatric* or paediatric* or "high school" or "secondary school" or school* or "young people" or "young person*" or "young adult*" or "young man" or "young men" or "young woman" or "young women" or student*)

2 AB=(emotion* AND (regulat* or dysregulat* or generat* OR modulat* OR adapt* OR cop* OR react* OR arous* OR avoid* OR behavio* OR suppress* OR ruminat*))

3 AB=(affect* AND (regulat* or dysregulat* or generat* OR modulat* OR adapt* OR cop* OR react* OR arous* OR avoid* OR behavio* OR suppress* OR ruminat*))

4 AB=(self-concept OR "mood regulat*" OR "situation selection" OR "situation modification" OR "attentional deployment" OR "response modulation" OR "problem focused coping" OR "impulsivity" OR hyper-reactivity OR "anger regulation" OR "experiential avoidance" OR "expressive suppression" OR "thought suppression" OR ruminat* OR "emotion regulation questionnaire" OR "Difficulties in Emotion Regulation Scale" OR "emotion regulation strategy")

5 #2 OR #3 OR #4

6 AB=(treat* or interven* or "early intervention" or "self-directed intervention" or "self-directed" or "public health")

7 AB=(phon* or mobile* or "smart-phone*" or device* or computer* or tablet* or "device-based" or "app-based" or apps or app or "sensor*" or "social media")

8 AB=((digital or virtual or online or interactive) and (health* or intervention or support or platform*))

9 AB=("eHealth" or "e-health" or "m-health" or mHealth or "tele-health" or telehealth)

10 #9 OR #8 OR #7

11 #10 AND #6 AND #5 AND #1

18 #17 and 2023 or 2022 or 2021 or 2020 or 2019 or 2000 or 2001 or 2002 or 2003 or 2004 or 2005 or 2006 or 2007 or 2008 or 2009 or 2010 or 2011 or 2012 or 2013 or 2014 or 2015 or 2016 or 2017 or 2018 (Publication Years) and English (Languages) and English (Languages) and Article (Document Types)

19 #10 AND #6 AND #5 AND #1 and 2023 or 2022 or 2021 or 2020 or 2019 or 2018 or 2017 or 2016 or 2015 or 2014 or 2013 or 2012 or 2011 or 2001 or 2002 or 2003 or 2004 or 2006 or 2007 or 2009 or 2010 or 2005 or 2008 or 1995 or 1996 or 1997 or 1998 or 1999 or 2000 (Publication Years) and Psychiatry or Public Environmental Occupational Health or Education Educational Research or Psychology Multidisciplinary or Psychology Clinical or Pediatrics or Health Care Sciences Services or Psychology Developmental or Psychology or Family Studies or Multidisciplinary Sciences or Nursing or Medicine Research Experimental or Medicine General Internal or Psychology Educational or Behavioral Sciences or Education Scientific Disciplines or Health Policy Services or Social Sciences Interdisciplinary or Social Work or Education Special or Psychology Social or Psychology Applied or Psychology Experimental or Sociology (Web of Science Categories) and

For peer review only

Book Review or Data Paper or Reprint or Editorial Material or Book Chapters or Biographical-Item or Review Article or Proceeding Paper or Early Access (Exclude – Document Types) and English (Languages) and Psychology or Psychiatry or Public Environmental Occupational Health or Health Care Sciences Services or Education Educational Research or Pediatrics or General Internal Medicine or Nursing or Family Studies or Science Technology Other Topics or Research Experimental Medicine or Behavioral Sciences or Social Sciences Other Topics or Social Work (Research Areas)

For peer review only

Database: Elsevier Embase 1947 to present

Search Strategy:

1	(adolescen* or juvenil* or youth* or teen* or pubescen* or pediatric* or paediatric* or "high school" or "secondary school" or school* or "young people" or "young person*" or "young adult*" or "young man" or "young men" or "young woman" or "young women" or student*).ti,ab
2	(emotion* AND (regulat* or dysregulat* or generat* OR modulat* OR adapt* OR cop* OR react* OR arous* OR avoid* OR behavio* OR suppress* OR ruminat*)).,ti,ab
3	(affect AND (regulat* or dysregulat* or generat* OR modulat* OR adapt* OR cop* OR react* OR arous* OR avoid* OR behavio* OR suppress* OR ruminat*)).,ti,ab
4	("self-concept" OR "mood regulat*" OR "situation selection" OR "situation modification" OR "attentional deployment" OR "response modulation" OR "problem focused coping" OR "impulsivity" OR "hyper-reactivity" OR "anger regulation" OR "experiential avoidance" OR "expressive suppression" OR "thought suppression" OR "ruminat*" OR "emotion regulation questionnaire" OR "Difficulties in Emotion Regulation Scale" OR "emotion regulation strategy").ti,ab
5	2 or 3 or 4
6	(treat* or interven* or "early intervention" or "self-directed intervention" or "self-directed" or "public health").ti,ab.
7	phon* or mobile* or "smart-phone*" or device* or computer* or tablet* or "device-based" or "app-based" or apps or app or "sensor*" or "social media").ti,ab.
8	((digital or virtual or online or interactive) and (health* or intervention or support or platform*)).ti,ab.
9	("eHealth" or "e-health" or "m-health" or mHealth or "tele-health" or telehealth).ti,ab.
10	7 or 8 or 9
11	1 and 5 and 6 and 10
**12**	**limit 11 to (human and english language and "remove medline records" and embase and yr="1995 -Current" and journal)**

For peer review only

Database: EThOS (PhD Theses)

Search Strategy:

1	"adolescen\" OR "high school" OR "secondary school" OR "youth" OR "young person" OR "young people"	Any word
2	AND "emotional regulation" OR "mood regulat\"	Any word
3	OR "emotion\" AND ("dysregulat\" OR "generat\" OR "modulat\" OR "adapt\" OR "cop\" OR react\ OR arous\ OR avoid\ OR behavio\ OR suppress\" OR "ruminat\")	Any word
4	OR affect AND (regulat\ or dysregulat\ or generat\ OR modulat\ OR adapt\ OR cop\ OR react\ OR arous\ OR avoid\ OR behavio\ OR "suppress\" OR "ruminat\”)	Any word
5	AND "prevent\" OR "promot\" OR "treat\" OR "evaluat\" OR "effective\" OR "interven\" OR "early intervention" OR "public health" OR "therap\"	Any word
6	AND "telemedicine\" OR "e-health\" OR "app-based" OR "virtual\" OR "web-based" OR "online\" OR "wearable" OR "device" OR "digital" OR "remote"	Any word

For peer review only

Database: OATD (PhD Theses)

Search Strategy:

1	"adolescen*" OR "youth*" OR "high school" OR "secondary school" OR "young person" OR "young people"	Any word
2	AND ("emotional regulation" OR "mood regulat*") OR ("emotion*" AND ("dysregulat*" OR "generat*" OR "modulat*" OR "adapt*" OR "cop*" OR react* OR arous* OR avoid* OR behavio* OR suppress*" OR "ruminat*")) OR (affect AND (regulat* or dysregulat* or generat* OR modulat* OR adapt* OR cop* OR react* OR arous* OR avoid* OR behavio* OR "suppress*" OR "ruminat*"))	Any word
3	AND "prevent*" OR "promot*" OR "treat*" OR "evaluat*" OR "effective*" OR "interven*" OR "early intervention" OR "public health" OR "therap*"	Any word
4	AND "telemedicine/" OR "e-health/" OR "app-based" OR "virtual*" OR "web-based" OR "online*" OR "wearable" OR "device" OR "digital" OR "remote"	Any word

For peer review only

Database: HMIC Health Management Information Consortium *1979 to November 2022*

Search Strategy:

----

1 exp adolescents/ or adolescent development/ or young adults/ or youth/

2 (adolescen* or juvenil* or youth* or teen* or pubescen* or pediatric* or paediatric* or "high school" or "secondary school" or school* or "young people" or "young person*" or "young adult*" or "young man" or "young men" or "young woman" or "young women" or student*).ti,ab.

3 1 or 2

4 "emotional regulation".mp.

5 (emotion* AND (regulat* or dysregulat* or generat* OR modulat* OR adapt* OR cop* OR react* OR arous* OR avoid* OR behavio* OR suppress* OR ruminat*)).ti,ab.

6 (affect AND (regulat* or dysregulat* or generat* OR modulat* OR adapt* OR cop* OR react* OR arous* OR avoid* OR behavio* OR suppress* OR ruminat*)).ti,ab

7 ("self-concept" OR "mood regulat*" OR "situation selection" OR "situation modification" OR "attentional deployment" OR "response modulation" OR "problem focused coping" OR impulsivity OR "hyper-reactivity" OR "anger regulation" OR "experiential avoidance" OR "behavio* avoidance" OR "expressive suppression" OR "thought suppression" OR ruminat* OR "emotion regulation questionnaire" OR "Difficulties in Emotion Regulation Scale" OR "emotion regulation strategy").ti,ab.

8 4 or 5 or 6 or 7

9 (prevent* or promot* or treat* or evaluat* or effective*).ti,ab.

10 (interven* or "early intervention" or "public health" or therap*).ti,ab.

11 9 or 10

12 telemedicine/ or e-health/

13 (phon* or mobile* or "smart-phone*" or device* or computer* or tablet* or "device-based" or "app-based" or apps or app or "sensor*" or "smart-home*" or "social media").ti,ab.

14 (tele and (phone* or nursing or health* or medicine or intervention or informatics or care or support or monitor* or presence or consultat* or rehab* or communicat*)).ti,ab.

15 (digital and (nursing* or health* or medicine or intervention or informatics or care or support or monitor* or presence or consultat* or rehab* or communicat* or platform*)).ti,ab.

16 (virtual and (nursing* or health* or medicine or intervention or informatics or care or support or monitor* or presence or consultat* or rehab* or communicat* or reality* or platform*)).ti,ab.

17 (online and (nursing* or health* or medicine or intervention or informatics or care or support or monitor* or presence or consultat* or rehab* or communicat* or platform*)).ti,ab

18	(interactiv* or "eHealth" or "e-health" or "e-therapy" or "e-learn" or "m-health").ti,ab.
19	("wireless health" or "health telematics" or "healthcare technology" or "therapeutic technology" or online or "medical informatics" or "medical information system" or "electronic health information" or "mobile application*" or "web-based" or website or wifi or "internet-delivered" or "internet-based" or "smart toy" OR “wearable*”).ti,ab.
20	12 or 13 or 14 or 15 or 16 or 17 or 18 or 19
21	3 and 8 and 11 and 20
**22**	**limit 21 to (English language and yr=”2000-2022”)**

For peer review only

Database: PsyArXiv

Search Strategy:

("emotional regulation" OR "mood regulat*" OR "emotion dysregulat*" OR "arous*" OR "avoid*" OR "behavio*" OR "suppress*" OR "ruminat*") AND ("prevent*" OR "promot*" OR "treat*" OR "evaluat*" OR "effective*" OR "interven*" OR "early intervention" OR "public health" OR "therap*") AND ("telemedicine" OR "e-health" OR "app-based" OR "virtual*" OR "web-based" OR "online*" OR "wearable" OR "device" OR "digital" OR "remote")

For peer review only

Database: TRIP

Search Strategy:

("adolescen*" OR "youth*" OR "high school" OR "secondary school" OR "young person" OR "young people") AND ("emotional regulation" OR "mood regulat*" OR "emotion dysregulat*" OR "arous*" OR "avoid*" OR "behavio*" OR "suppress*" OR "ruminat*") AND ("prevent*" OR "promot*" OR "treat*" OR "evaluat*" OR "effective*" OR "interven*" OR "early intervention" OR "public health" OR "therap*") AND ("telemedicine" OR "e-health" OR "app-based" OR "virtual*" OR "web-based" OR "online*" OR "wearable" OR "device" OR "digital" OR "remote")

For peer review only

Database: ClinicalTrials.gov

Search Strategy:

("telemedicine" OR "e-health" OR "virtual*" OR "digital") | Emotional Regulation | Child

Database: ACM Digital Library

Search Strategy:

[[All: "emotional regulation"] OR [All: "mood regulat*"] OR [All: "emotion dysregulat*"]] AND [[All: "prevent*"] OR [All: "promot*"] OR [All: "treat*"] OR [All: "evaluat*"] OR [All: "effective*"] OR [All: "interven*"] OR [All: "early intervention"] OR [All: "public health"] OR [All: "therap*"]] AND [[All: "adolescen*"] OR [All: "youth*"] OR [All: "high school"] OR [All: "secondary school"] OR [All: "young person"] OR [All: "young people"]] AND [[All: "app-based"] OR [All: "virtual*"] OR [All: "web-based"] OR [All: "online*"] OR [All: "digital"] OR [All: "remote"]]

For peer review only

PRISMA-P (Preferred Reporting Items for Systematic review and Meta-Analysis Protocols) 2015 checklist: recommended items to address in a systematic review protocol*

Section and topic		Item No	Checklist item
**ADMINISTRATIVE INFORMATION**			
Title:			
Identification	✓	1a	Identify the report as a protocol of a systematic review
Update	N/A	1b	If the protocol is for an update of a previous systematic review, identify as such
Registration	✓	2	If registered, provide the name of the registry (such as PROSPERO) and registration number
Authors:			
Contact	✓	3a	Provide name, institutional affiliation, e-mail address of all protocol authors; provide physical mailing address of corresponding author
Contributions	✓	3b	Describe contributions of protocol authors and identify the guarantor of the review
Amendments	✓	4	If the protocol represents an amendment of a previously completed or published protocol, identify as such and list changes; otherwise, state plan for documenting important protocol amendments
Support:			
Sources	✓	5a	Indicate sources of financial or other support for the review
Sponsor	✓	5b	Provide name for the review funder and/or sponsor
Role of sponsor or funder	✓	5c	Describe roles of funder(s), sponsor(s), and/or institution(s), if any, in developing the protocol
**INTRODUCTION**			
Rationale	✓	6	Describe the rationale for the review in the context of what is already known
Objectives	✓	7	Provide an explicit statement of the question(s) the review will address with reference to participants, interventions, comparators, and outcomes (PICO)
**METHODS**			
Eligibility criteria	✓	8	Specify the study characteristics (such as PICO, study design, setting, time frame) and report characteristics (such as years considered, language, publication status) to be used as criteria for eligibility for the review
Information sources	✓	9	Describe all intended information sources (such as electronic databases, contact with study authors, trial registers or other grey literature sources) with planned dates of coverage
Search strategy	✓	10	Present draft of search strategy to be used for at least one electronic database, including planned limits, such that it could be repeated
Study records:			
Data management	✓	11a	Describe the mechanism(s) that will be used to manage records and data throughout the review

For peer review only

Selection process	✓	11b	State the process that will be used for selecting studies (such as two independent reviewers) through each phase of the review (that is, screening, eligibility and inclusion in meta-analysis)
Data collection process	✓	11c	Describe planned method of extracting data from reports (such as piloting forms, done independently, in duplicate), any processes for obtaining and confirming data from investigators
Data items	✓	12	List and define all variables for which data will be sought (such as PICO items, funding sources), any pre-planned data assumptions and simplifications
Outcomes and prioritization	✓	13	List and define all outcomes for which data will be sought, including prioritization of main and additional outcomes, with rationale
Risk of bias in individual studies	✓	14	Describe anticipated methods for assessing risk of bias of individual studies, including whether this will be done at the outcome or study level, or both; state how this information will be used in data synthesis
Data synthesis	✓	15a	Describe criteria under which study data will be quantitatively synthesised
	✓	15b	If data are appropriate for quantitative synthesis, describe planned summary measures, methods of handling data and methods of combining data from studies, including any planned exploration of consistency (such as I^2, Kendall's τ)
	✓	15c	Describe any proposed additional analyses (such as sensitivity or subgroup analyses, meta-regression)
	✓	15d	If quantitative synthesis is not appropriate, describe the type of summary planned
Meta-bias(es)	✓	16	Specify any planned assessment of meta-bias(es) (such as publication bias across studies, selective reporting within studies)
Confidence in cumulative evidence	✓	17	Describe how the strength of the body of evidence will be assessed (such as GRADE)

*** It is strongly recommended that this checklist be read in conjunction with the PRISMA-P Explanation and Elaboration (cite when available) for important clarification on the items. Amendments to a review protocol should be tracked and dated. The copyright for PRISMA-P (including checklist) is held by the PRISMA-P Group and is distributed under a Creative Commons Attribution Licence 4.0.**

From: Shamseer L, Moher D, Clarke M, Ghersi D, Liberati A, Petticrew M, Shekelle P, Stewart L, PRISMA-P Group. Preferred reporting items for systematic review and meta-analysis protocols (PRISMA-P) 2015: elaboration and explanation. BMJ. 2015 Jan 2;349(jan02 1):g7647.

For peer review only
